# Analysis of straight conjugate internal gear pump through numerical modeling and experimental validation

**DOI:** 10.1371/journal.pone.0270979

**Published:** 2022-07-28

**Authors:** Hongqiang Chai, Guolai Yang, Guoguo Wu, Guixiang Bai, Chuanchuan Cao

**Affiliations:** 1 College of Energy and Power Engineering, Lanzhou University of Technology, Lanzhou, Gansu, China; 2 School of Intelligent Manufacturing Engineering, Chongqing University of Arts and Sciences, Chongqing, China; University of Vigo, SPAIN

## Abstract

As a medium and low pressure gear machine without automatic compensation structure for axial and radial clearances, the friction pairs in the straight conjugate internal gear pumps (SCIGPs) depend on the fixed small clearances to seal, lubricate and transfer the force. The oil film design of the friction pairs has become an important part of gear pump design. However, there has never been a publicly published research on the oil film design of the SCIGP in past literature. This paper applies orthogonal test to the oil film design of the SCIGP for the first time to determine the best working clearances. With this goal, the paper first provides the mathematical models for analyzing the internal leakage flow and the viscous friction loss, which elucidate the relationships between the leakage and the friction loss with working conditions. After that, the orthogonal test scheme for numerical simulation was designed on the basis of determining the range of oil film thickness. The paper also propounds the viewpoints of using the range-method to estimate the primary and secondary relationship of factors and determining the optimal combination according to the test target. Based on this concept, the main factors affecting the target are procured and the optimal working clearances of the friction pairs are determined. For the purpose of verifying the model, the redesigned prototype was tested and compared with the simulation results. The results validate the applicability of the simulation model and the correctness of the simulation method. Finally, the paper summarizes the ways to improve the total efficiency and the working conditions at the highest efficiency.

## 1. Introduction

Positive displacement pumps are the power component of hydraulic system, and their overall performance determine the life and the reliability of hydraulic products. As one of the three major hydraulic pumps, gear pumps, especially internal gear pumps, have the advantages of compact structure, low wear, stable operation and strong anti-pollution ability, etc. They are widely used in construction machinery, injection molding machinery and metallurgical machinery and other fields [1–4].

The gear pairs are the only moving parts in the gear pumps, and their tooth profile curves affect the key performance of the pumps. The SCIGP is a new gear pump developed after the involute and the cycloid internal gear pumps. Its moving parts consist of an external gear with a straight tooth profile and an internal gear ring with a high-order arc tooth profile [5]. As a medium and low pressure gear pump, there is no automatic compensation structure for axial and radial clearances inside the pump. The friction pairs depend on the fixed small clearances to achieve sealing, lubrication and transmission of force. Wei et al. used the general tooth profile normal reversal method to solve the conjugate tooth profile curves [6]. Afterwards, Hu et al. optimized the tooth profile parameters on this basis and established an optimal design mathematical model [7].

Like other positive displacement pumps, the SCIGPs generally have the characteristic of large outlet flow fluctuation. This is a prejudicial feature. The pressure pulsation caused by the combination of pulsating flow and system loop impedance not only damages the weak parts in the system, but also causes component vibration and fluid noise [8–11], etc. For the cause of flow unevenness, the author’s research team based on the combination of volume change method and numerical verification elicited that the uneven internal leakage is the main factor of flow pulsation, and the influence of geometric flow pulsation rate is small [12]. Since the experiment was carried out under no-load conditions, the influence of oil compressibility on flow pulsation was not considered. The approximate formula of flow pulsation rate was first proposed by Cui et al. [13], but its application range was limited due to accuracy problems.

The lumped parameter model based on the flow continuity equations can quickly attain the pump flow characteristics. For example, Rundo et al. proposed a lumped parameter model that can estimate the steady-state flow-pressure characteristics [8, 14]. Du et al. analyzed the working performance of the pump through a lumped parameter model [15], etc. However, the researches based only on the movement flow ignore the influence of fluid viscosity and compressibility on pump operation, and cannot reflect the full movement space of fluid particles.

The pressure shock and the cavitation in the process of trapped oil seriously affect the working stability and the life of gear pumps [16]. Wang et al. exerted the tangent polar coordinate method to procure the expression of the change of trapped oil volume [17], but it did not reflect the change of trapped oil pressure. Sedri et al. set up a new set of unloading grooves on the external gears that meshed with each other to eliminate the trapped oil phenomenon [18]. This structure did not consider the leakage between the meshing tooth surfaces and the gears strength. Other research contents include: Duan et al. deduced the calculation formula of gear pairs coincidence degree, and studied the relationship between gear geometric parameters and coincidence degree [19, 20].

If the oil film between the friction pairs is too thin or cannot be formed, the friction pairs will be worn or burned. If the oil film is too thick, it will not be able to seal, and it will not even be able to establish load-adapted pressure. The oil film design of the friction pairs has become an important part of the fluid power design. To date, no one has published any relevant research for SCIGPs. In view of this, this paper applies the orthogonal test to the oil film design of the SCIGP for the first time to determine the best working clearances. First, the paper systematically expounds the mathematical models of internal leakage and viscous friction loss, and ascertains the value range of the oil film thickness according to the characteristics of the hydrostatic support (Section 2). On this basis, an orthogonal test design is carried out and a CFD simulation model is established (Section 3). After that, the paper uses the orthogonal test analysis table to execute numerical simulation. Finally, the paper introduces the experimental activities of the prototype. In particular, the experimental and simulation results are compared, and the verification results are shown in this section (Section 4).

## 2. SCIGP modeling: Geometric modeling

### 2.1 Selection of oil film thickness

There are five pairs of friction pairs in the SCIGP (i.e., the friction pair between the gear end face and the shell, the friction pair between the addendum and the crescent diaphragm, the friction pair between the outer wall of the internal ring gear and the shell, the friction pair between the inner wall of the external gear and the bearing, and the friction pair between the meshing tooth surfaces). The value range of the working clearances (i.e., oil film thicknesses) of friction pairs can be determined by the hydrostatic support oil film theory.

**Fig 1:**
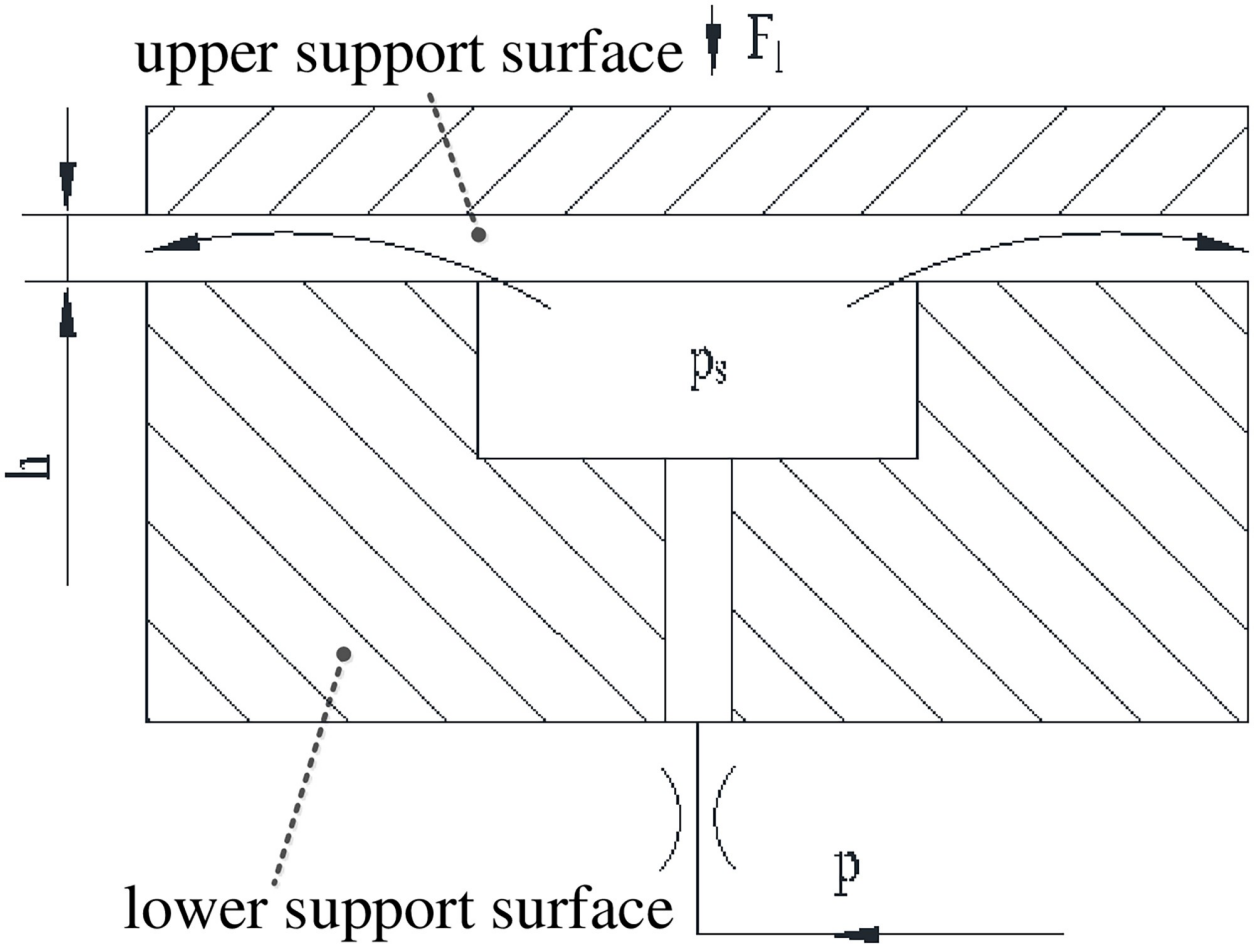
Typical structure of hydrostatic support.

The hydrostatic support oil film is a series combination of fixed damping and variable clearance damping of support surface [21], and the typical structure is shown in Fig 1.

In Fig 1, the pressure-flow characteristic equation of any fixed damper can be expressed as:

$$\Delta p^{\prime }=R_{\text{f}}Q$$ (1)

The pressure-flow characteristic equation of variable clearance damping with any shape can be expressed as:

$$Q=k_{q}h^{3}\Delta p^{\prime \prime }$$ (2)

The oil with pressure Ps flows into the low pressure cavity after variable clearance, and assuming that the pressure is 0 in it. Δ*p*′ = *p* −*p*_*s*_ and Δ*p*″ = *p*_*s*_ − 0 = *p*_*s*_ are Substituted into (1) and (2) to procure:

$$p_{\text{s}}=\frac{p}{1+R_{\text{f}}k_{q}h^{3}}$$ (3)

Assuming *K* = *R*_*f*_*k*_*q*_, then (3) can be expressed as:

$$p_{\text{s}}=\frac{p}{1+Kh^{3}}$$ (4)

Considering that the input pressure *p* of hydrostatic support is variable, and the dimensionless pressure ratio *α* = *p*_*s*_/*p* is brought in (4) to procure:

$$\alpha =\frac{1}{1+Kh^{3}}$$ (5)

**Fig 2:**
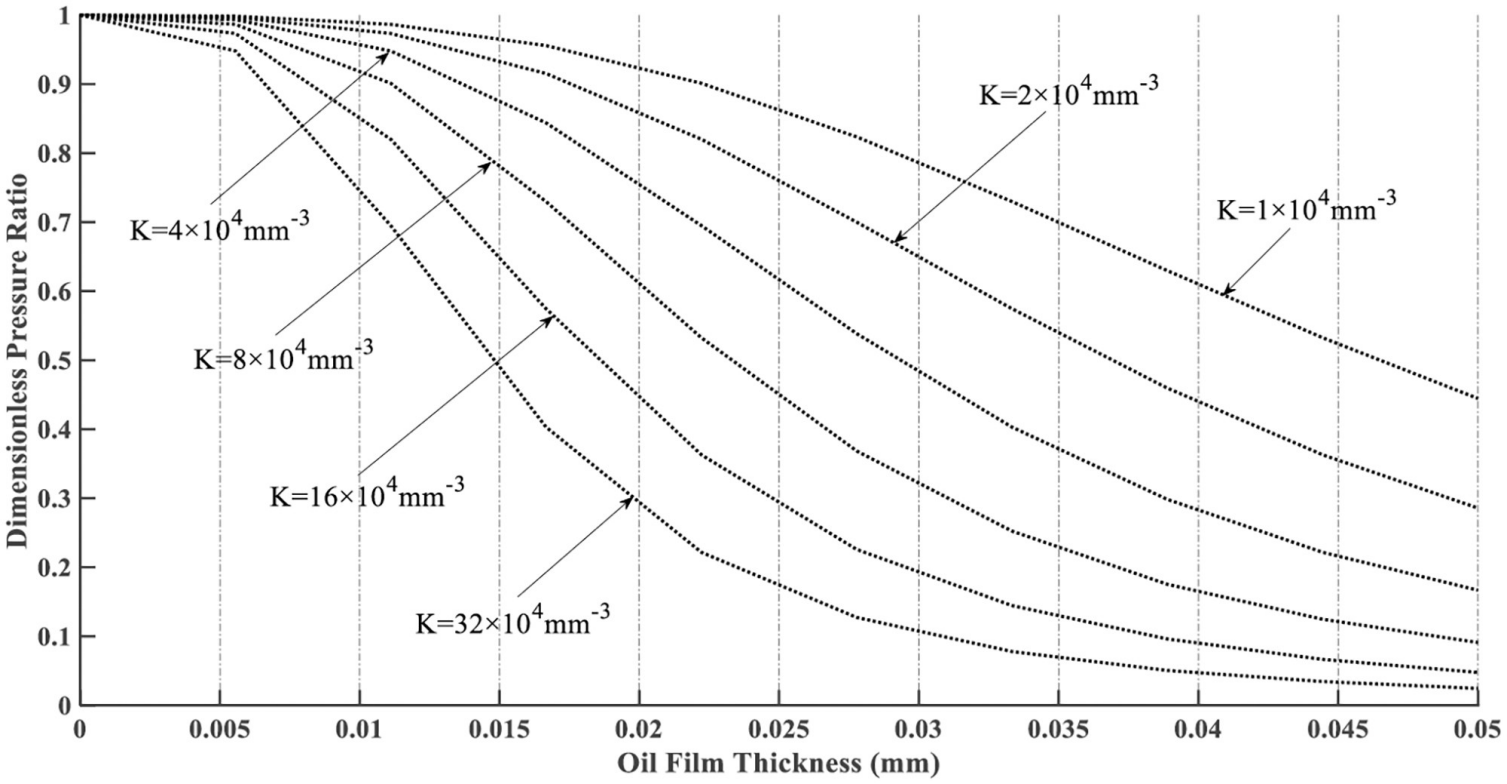
Working characteristic curves of the hydrostatic support.

Eq (5) is called the characteristic equation of hydrostatic support. According to (5), the working characteristic curves under different structural parameters can be procured, as shown in Fig 2.

Fig 2 shows that the larger the value of *K*, the steeper the curves, indicating that a small change of *h* will bring about a significant change of *α*. This is very beneficial for adapting to external loads and improving the bearing capacity of the new oil film support surface. It can also be seen from the curves that when the oil film thickness is less than 0.01mm and greater than 0.04mm, each curve is relatively flat, that is, the ability to adapt to external load changes is not strong. Therefore, the range of oil film thickness is 0.01–0.04 mm.

### 2.2 Mathematical model of internal leakage

**Fig 3:**
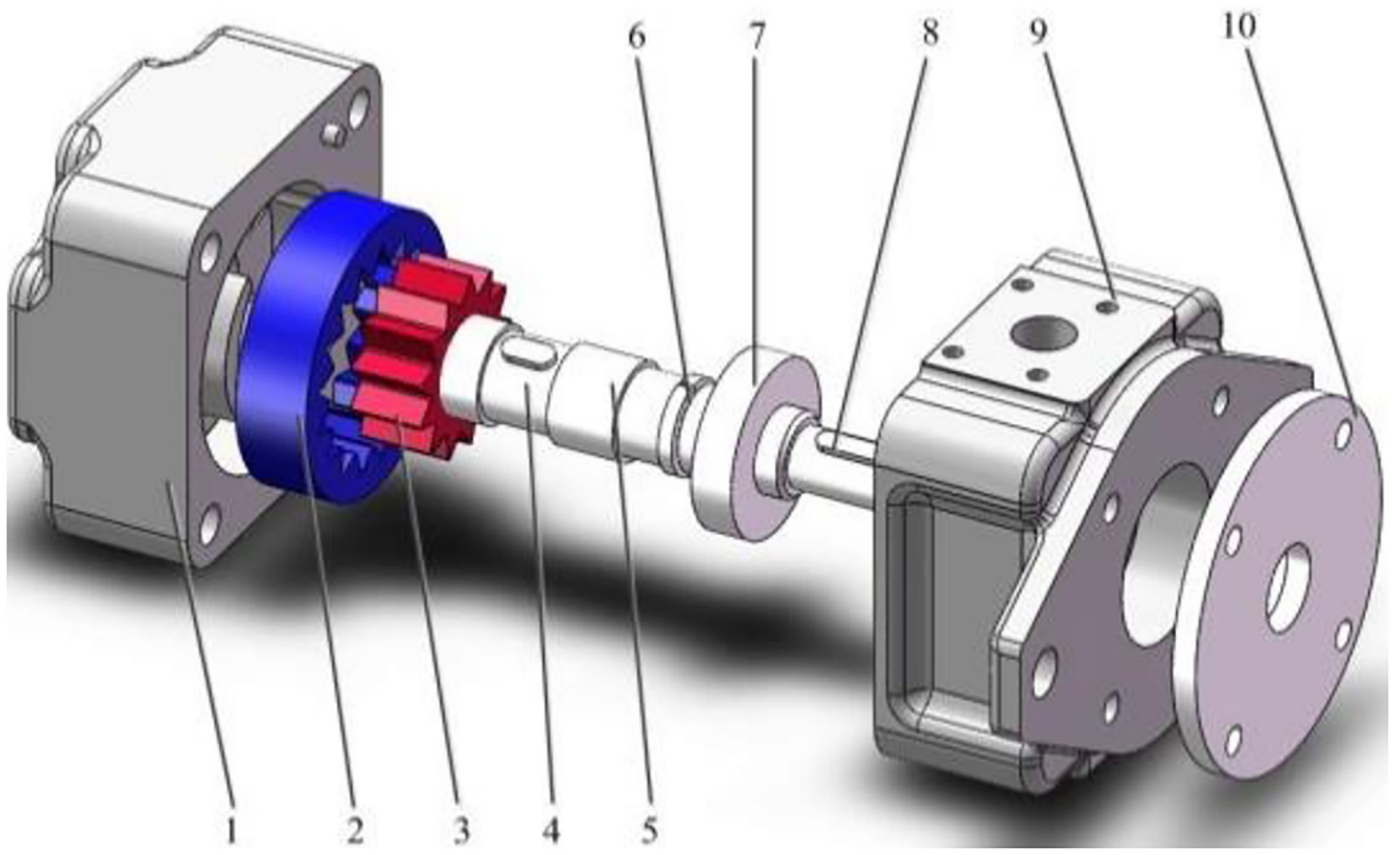
Exploded view of three-dimensional assembly. 1-pump body; 2-internal gear ring; 3-external gear; 4-gear shaft; 5-sliding bearing; 6-bearing retaining ring; 7-rolling gearing; 8-ordinary flat key; 9-pump cover; 10-bearing cover.

In order to observe the internal structure of the SCIGP more intuitively, an exploded view of the assembly is established, as shown in Fig 3.

**Fig 4:**
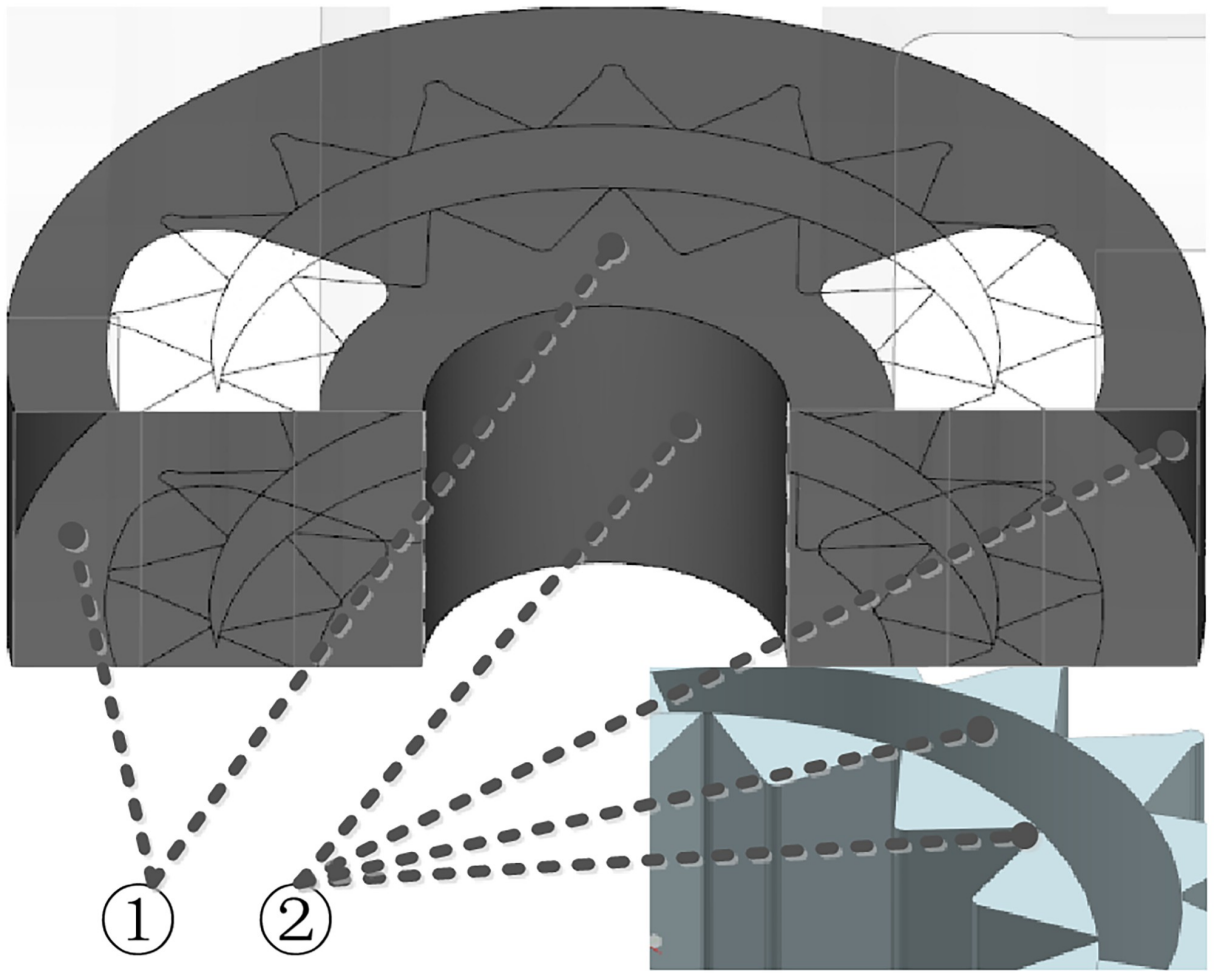
Solid model of internal leakage. ①Axial clearance ②Radial clearance.

The internal leakage of the SCIGP mainly includes three ways: axial clearance leakage, radial clearance leakage and tooth surface contact leakage. In the case of normal meshing, the leakage through the tooth surface contact is very small, so it is not considered. The solid model of the internal leakage is established, as shown in Fig 4.

Fig 4 shows that the axial leakage includes two leakages on the upper and lower end faces of gear pair, and the radial leakage includes four leakages at the internal and external cylindrical surfaces of gear pair, and the external gear and internal gear ring addendums.

#### 2.2.1 Axial clearance leakage flow Δ*q*_1_

**Fig 5:**
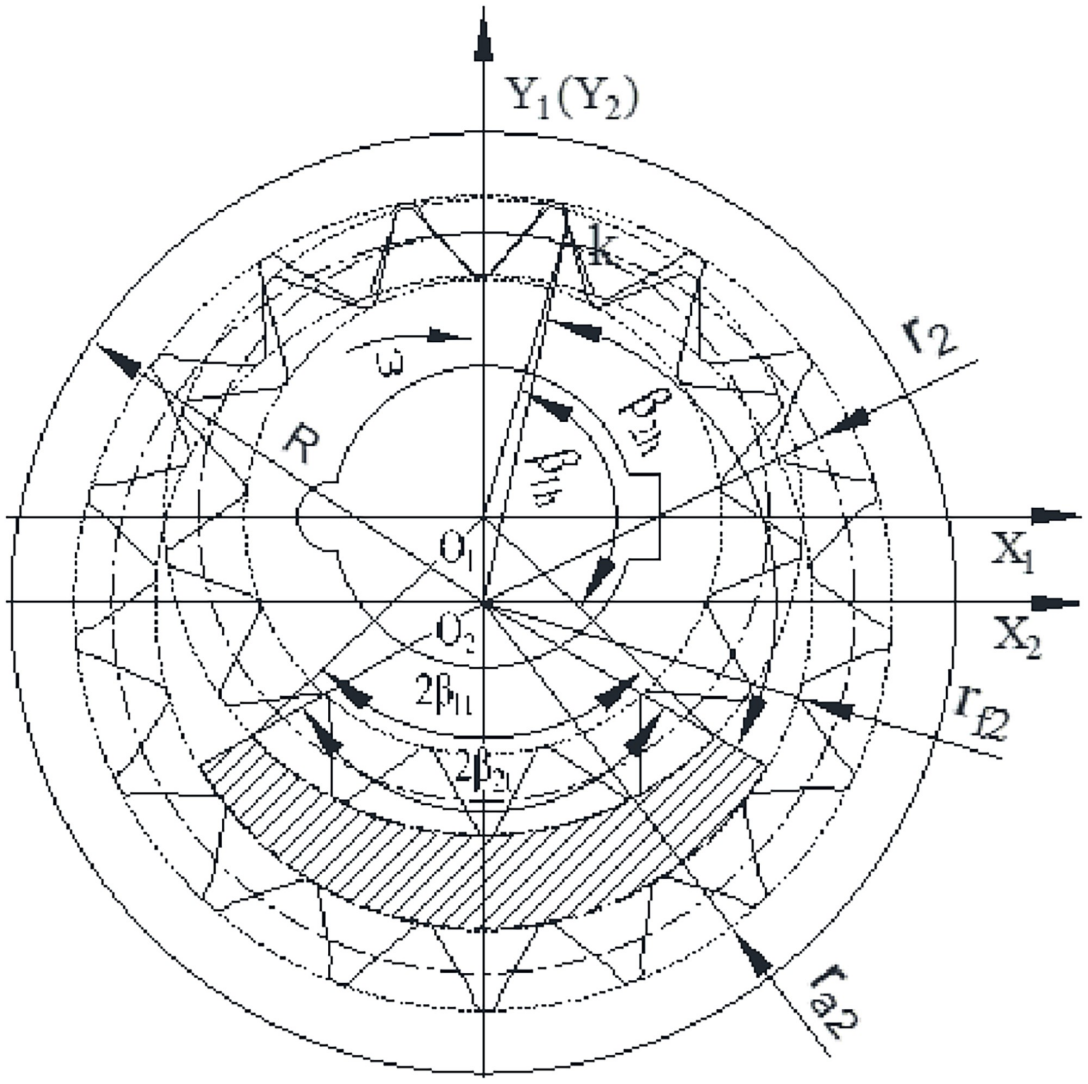
Schematic diagram of regional division on the gear end face.

Axial clearance leakage can be calculated according to the clearance flow theory between two parallel discs, without considering the oil flow in the end clearance caused by gear motion. In order to facilitate the calculation of leakage flow, the gear end face is divided into three parts—i.e., the section in contact with the low pressure cavity, the section in contact with the high pressure cavity, and the transition section between the high and low pressure cavities, as shown in Fig 5. The pressure in the transition region follows the step distribution law, which can be approximately considered to be linear. Therefore, the pressure difference in the transition region is assumed to be Δ*p*/2. According to the pressure difference-flow formula between the parallel discs and combining with Fig 5, the total leakage Δ*q*_1_ in the gear end face clearance can be procured.


$$\Delta q_{1}=\frac{h_{1}^{3}\Delta p}{\mu }\left[\frac{\beta_{1h}+\beta_{1\text{t}}}{6\text{ln}(r_{\text{f}1}/r)}+\frac{\beta_{2h}+\beta_{2\text{t}}}{6\text{ln}(R/r_{\text{f2}})}\right]$$ (6)

Assuming $k_{q1}=\frac{\beta_{1h}+\beta_{1\text{t}}}{6\text{ln}(r_{\text{f}1}/r)}+\frac{\beta_{2h}+\beta_{2\text{t}}}{6\text{ln}(R/r_{\text{f2}})}$, the (6) can be expressed as:

$$\Delta q_{1}=\frac{k_{q1}h_{1}^{3}\Delta p}{\mu }$$ (7)

#### 2.2.2 Radial clearance leakage flow Δ*q*_2_

*1) Leakage flow between addendum and crescent diaphragm*.

**Fig 6:**
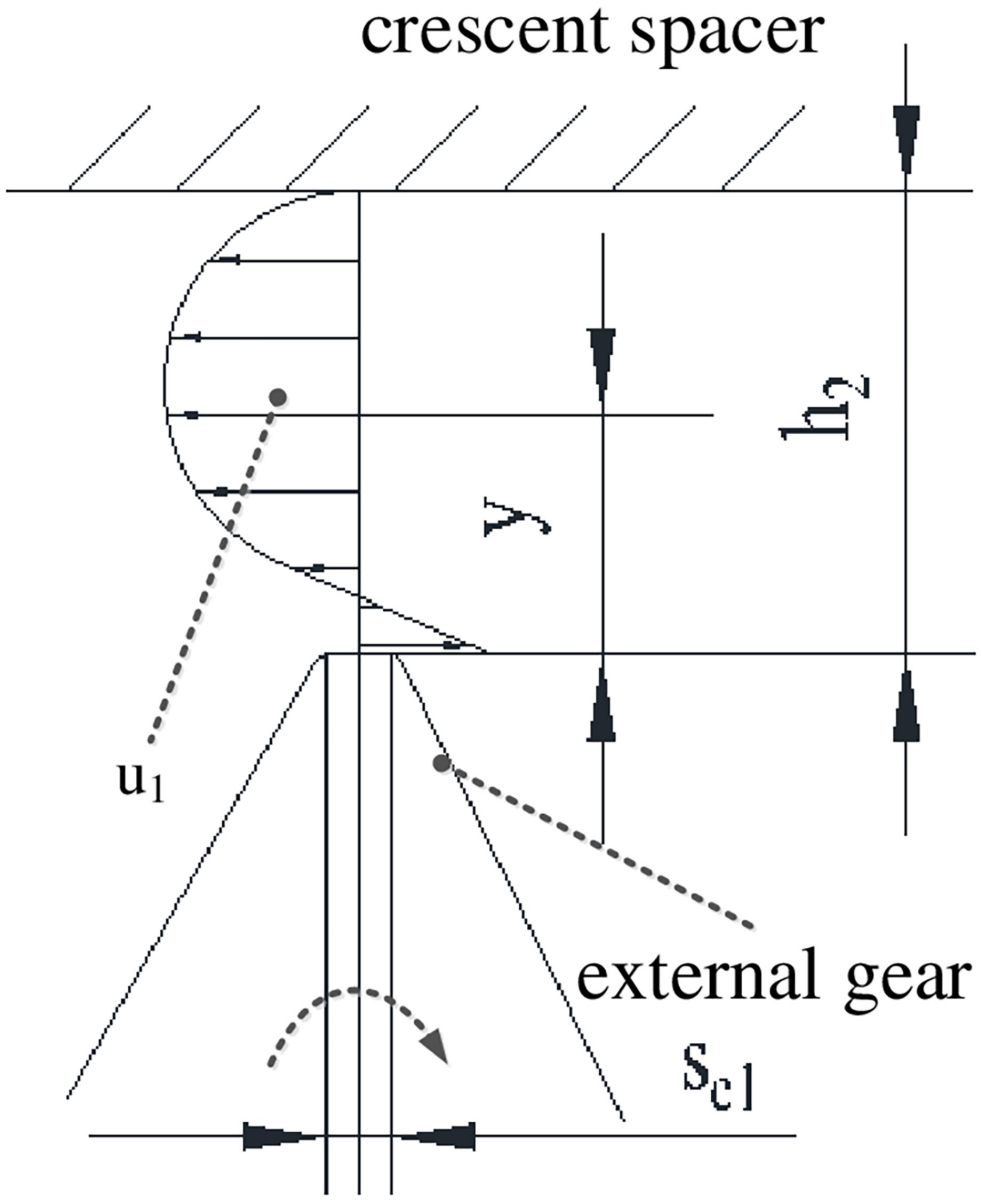
Distribution diagram of fluid velocity between external gear addendum and crescent diaphragm.

Since the oil film thickness in the addendum clearance is much smaller than the diameter of the cylinder, the laminar flow motion is calculated according to the theory of parallel plate clearance flow. On the one hand, the oil on the addendum flows along the opposite direction as the rotational motion under the action of pressure difference $\Delta p/{z^{\prime }}_{1}$ between the two sides of gear teeth, and its velocity is parabolic distribution. On the other hand, it flows along the same direction due to the frictional traction of gear teeth, and its speed is linearly distributed. The velocity superposition results of differential pressure flow and shear flow are shown in Fig 6.

In Fig 6, the total leakage velocity *u*_1_ between external gear addendum and crescent diaphragm can be expressed as:

$$u_{1}=\frac{\Delta p/{z^{\prime }}_{1}}{2\mu s_{e\text{1}}}(h_{2}y-y^{2})-v_{\text{a}1}\left(1-\frac{y}{h_{2}}\right)$$ (8)

Among them, the linear velocity of external gear addendum is $v_{\text{a}1}=\frac{\pi n_{1}r_{\text{a}1}}{30}$, so that the leakage flow Δ*q*_*h*2_ between external gear addendum and crescent diaphragm can be procured.


$${\Delta q}_{h\text{2}}=\frac{B\Delta p}{12\mu s_{e\text{1}}{z^{\prime }}_{1}}h_{2}^{3}-\frac{B\pi n_{1}r_{\text{a}1}}{60}h_{2}$$ (9)

In the same way, the leakage flow Δ*q*_*h*3_ between internal gear ring addendum and crescent diaphragm can be procured.


$${\Delta q}_{h\text{3}}=\frac{B\Delta p}{12\mu s_{e\text{2}}{z^{\prime }}_{2}}h_{3}^{3}-\frac{B\pi n_{2}r_{\text{a}1}}{60}h_{3}$$ (10)

*2) Leakage flow between the inner wall of external gear and the bearing*

Due to the uneven force on the gear shaft, it is eccentric with the bearing. Moreover, there is no relative movement between the inner wall of external gear and the sliding bearing. Therefore, the radial clearance flow is calculated according to the eccentric annular seam. Since the pressure difference of the annular seam is very small, it is assumed to be Δ*p*/8. Thus, the leakage flow Δ*q*_*h*4_ in the eccentric annular seam can be procured.


$$\Delta q_{h\text{4}}=\left(1+1.5\varepsilon^{2}\right)\frac{\pi dh_{4}^{3}}{96\mu e}\Delta p$$ (11)

*3) Leakage flow between the outer wall of internal gear ring and the inner wall of the shell*

The radial clearance leakage between the outer wall of internal gear ring and the inner wall of the shell can be calculated according to the theory of parallel plate clearance flow. The leakage flow Δ*q*_*h*5_ can be procured.


$${\Delta q}_{h\text{5}}=\frac{B\Delta p}{24\pi \mu R}h_{5}^{3}-\frac{BR\pi n_{2}}{60}h_{5}$$ (12)

In summary, the total leakage flow Δ*q* inside gear pump can be procured.


$$\Delta q=\Delta q_{1}+\Delta q_{2}=\frac{B\Delta p}{24\mu }\left(\frac{2h_{2}^{3}}{s_{e\text{1}}{z^{\prime }}_{1}}+\frac{2h_{3}^{3}}{s_{e\text{2}}{z^{\prime }}_{2}}+\frac{h_{5}^{3}}{\pi R}\right)-\frac{B\pi }{60}\left(n_{1}r_{\text{a}1}h_{2}+n_{2}r_{\text{a2}}h_{3}+Rn_{2}h_{5}\right)+\frac{\Delta p}{96\mu }\left[\left(1+1.5\varepsilon^{2}\right)\frac{\pi dh_{4}^{3}}{e}+96k_{q1}h_{1}^{3}\right]$$ (13)

Eq (13) shows that the greater the pressure difference, the smaller the viscosity, and the higher the leakage flow; the higher the speed, the lower the leakage flow.

### 2.3 Mathematical model of power loss

The leakage flow equations are systematically expounded above, and the power loss caused by them is clear, which is not explained here. Next, the power loss caused by viscous friction in the working clearance is discussed in detail.

#### 2.3.1 Axial clearance power loss Δ*N*_1_

**Fig 7:**
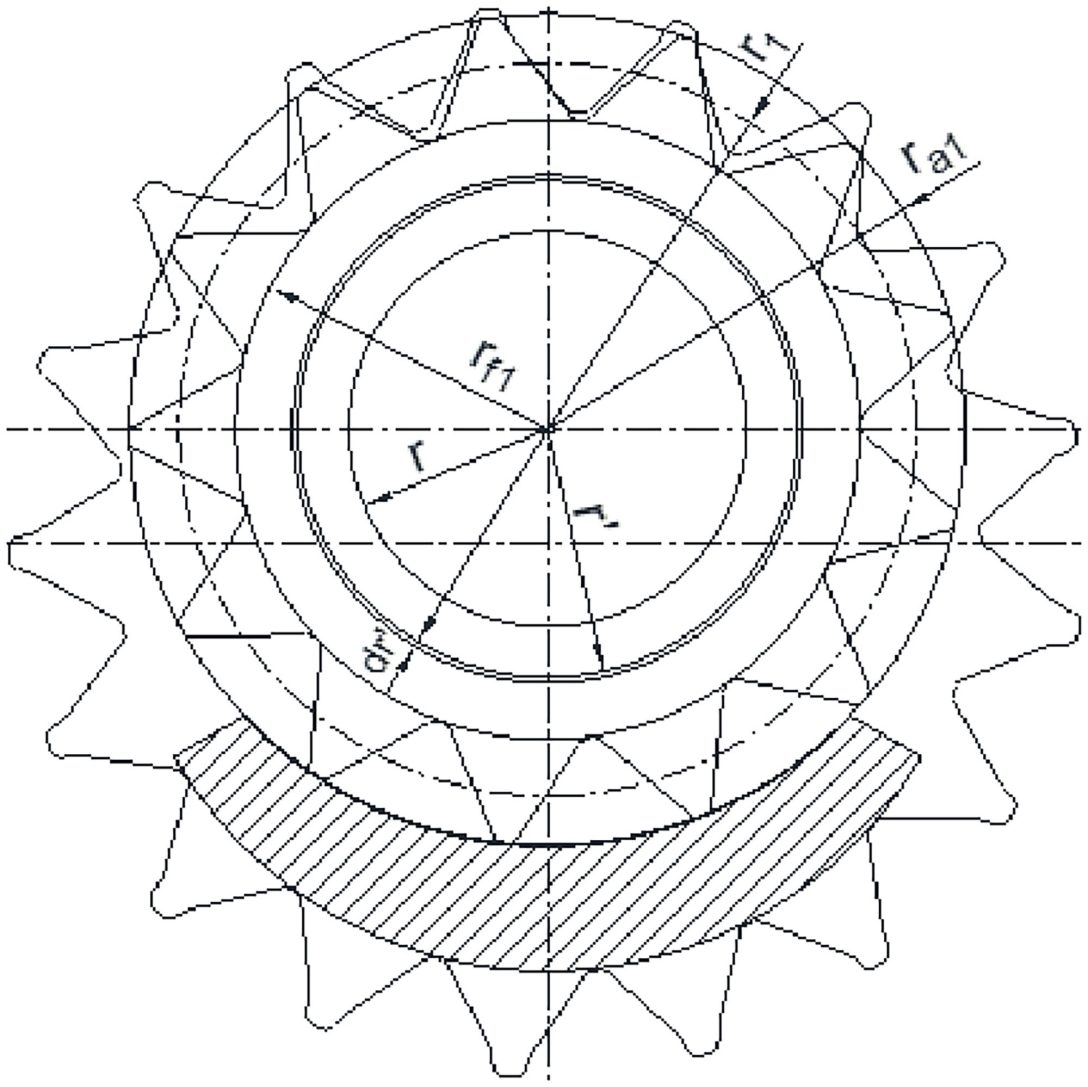
Simplified model of viscous friction on the end face of external gear.

The simplified model of viscous friction on the end face of external gear is established, as shown in Fig 7. Then the linear velocity *v*_1_ of oil at the radius ${r^{\prime }}_{1}$ is:

$$v_{1}=\frac{\pi n_{1}{r^{\prime }}_{1}}{30}$$ (14)

From Newton’s friction theorem, the friction shear stress *τ*_*h*1_ of oil on the end face of external gear can be procured.


$$\tau_{h\text{1}}=\mu \frac{\pi n_{1}{r^{\prime }}_{1}}{30h_{1}}$$ (15)

Because the micro-ring area with the width *dr*′is $dA^{\prime }=2\pi {r^{\prime }}_{1}dr^{\prime }$, the oil viscous friction loss $\Delta {N^{\prime }}_{\text{f}1}$ on the unilateral end face from the gear shaft to the dedendum circle can be procured.


$$\Delta {N^{\prime }}_{\text{f}1}=\int \tau_{h1}dA^{\prime }v_{1}=\frac{\mu \pi^{3}n_{1}^{2}}{1800h_{1}}(r_{\text{f}1}^{4}-r^{4})$$ (16)

Considering that the part involved in friction on the unilateral end face from the dedendum circle to the addendum circle is only gear teeth. Therefore, in the calculation of the oil viscous friction loss, it can be approximately considered that the areas of tooth thickness and between the teeth are equal. In this way, the oil viscous friction loss $\Delta {N^{\prime \prime }}_{\text{f1}}$ on the unilateral end face from the dedendum circle to the addendum circle can be procured.


$$\Delta {N^{\prime \prime }}_{\text{f1}}=\frac{\mu \pi^{3}n_{1}^{2}r_{1}}{2700h_{1}}(r_{\text{a}1}^{3}-r_{\text{f}1}^{3})$$ (17)

Thereby, the oil viscous friction loss Δ*N*_f1_ in the end face clearance of external gear can be procured.


$$\Delta N_{\text{f1}}=\frac{\mu \pi^{3}n_{1}^{2}}{50h_{1}}\left[\frac{1}{18}(r_{\text{f}1}^{4}-r^{4})+\frac{1}{27}r_{1}(r_{\text{a}1}^{3}-r_{\text{f}1}^{3})\right]$$ (18)

In the same way, the oil viscous friction loss Δ*N*_f2_ in the end face clearance of internal ring gear can be procured.


$$\Delta N_{\text{f2}}=\frac{\mu \pi^{3}n_{2}^{2}}{50h_{1}}\left[\frac{1}{18}(R^{4}-r_{\text{f2}}^{4})+\frac{1}{27}r_{2}(r_{\text{f2}}^{3}-r_{\text{a2}}^{3})\right]$$ (19)

#### 2.3.2 Radial clearance power loss Δ*N*_2_

There is no relative movement between the inner wall of external gear and the bearing, so it is only necessary to calculate the viscous friction loss between the addendums of external gear and internal gear ring and the crescent diaphragm, and the viscous friction loss between the outer wall of internal gear ring and the shell.

The derivative of both ends of (8) for any height *y* can be procured:

$$\frac{du_{1}}{dy}=\frac{\Delta p}{2\mu s_{e1}{z^{\prime }}_{1}}(h_{2}-2y)+\frac{\pi n_{1}r_{\text{a}1}}{30h_{2}}$$ (20)

The oil friction shear stress *τ*_*h*2_ on the external gear addendum surface can be procured by Newton’s friction theorem.


$$\tau_{h2}=\frac{\Delta ph_{2}}{2s_{e1}{z^{\prime }}_{1}}+\frac{\mu \pi n_{1}r_{\text{a}1}}{30h_{2}}$$ (21)

Since the friction area of external gear addendum is $A_{1}={z^{\prime }}_{1}Bs_{e1}$, the oil viscous friction loss Δ*N*_*h*2_ can be procured.


$${\Delta N}_{h2}=\frac{1}{60}B\pi n_{1}r_{\text{a}1}{z^{\prime }}_{1}s_{e1}\left(\frac{\Delta ph_{2}}{{z^{\prime }}_{1}s_{e1}}+\frac{\mu \pi n_{1}r_{\text{a}1}}{15h_{2}}\right)$$ (22)

In the same way, the oil viscous friction loss Δ*N*_*h*3_ on the internal gear ring addendum surface can be procured.


$${\Delta N}_{h3}=\frac{1}{60}B\pi n_{2}r_{\text{a}2}{z^{\prime }}_{2}s_{e\text{2}}\left(\frac{\Delta ph_{3}}{{z^{\prime }}_{2}s_{e\text{2}}}+\frac{\mu \pi n_{2}r_{\text{a}2}}{15h_{3}}\right)$$ (23)

Further, the oil viscous friction loss Δ*N*_*h*5_ on the outer wall of internal gear ring can be procured.


$${\Delta N}_{h\text{5}}=\frac{1}{30}B\pi n_{2}R\left(\frac{\Delta ph_{5}}{2}+\frac{\mu n_{2}\pi^{2}R^{2}}{15h_{5}}\right)$$ (24)

In summary, the total viscous friction loss Δ*N* inside gear pump can be procured.


$$\begin{array}{l} \Delta N=\Delta N_{1}\text{+}\Delta N_{2}=\frac{\mu \pi^{3}}{50h_{1}}\frac{n_{1}^{2}}{18}\left[\left(r_{\text{f}1}^{4}-r^{4})+\frac{n_{1}^{2}}{27}r_{1}(r_{\text{a}1}^{3}-r_{\text{f}1}^{3})\text{+}\frac{n_{2}^{2}}{18}(R^{4}-r_{\text{f2}}^{4})+\frac{n_{2}^{2}}{27}r_{2}(r_{\text{f2}}^{3}-r_{\text{a2}}^{3}\right)\right]\text{+}\frac{1}{60}B\pi n_{1}r_{\text{a}1}{z^{\prime }}_{1}s_{e1}\left(\frac{\Delta ph_{2}}{{z^{\prime }}_{1}s_{e1}}+\frac{\mu \pi n_{1}r_{\text{a}1}}{15h_{2}}\right)\text{+}\ldots \\ \;\;\;\frac{1}{60}B\pi n_{2}\;\left[r_{\text{a2}}{z^{\prime }}_{2}s_{e2}\left(\frac{\Delta ph_{3}}{{z^{\prime }}_{2}s_{e2}}+\frac{\mu \pi n_{2}r_{\text{a2}}}{15h_{3}}\right)\text{+2}R\left(\frac{\Delta ph_{5}}{2}+\frac{\mu n_{2}\pi^{2}R^{2}}{15h_{5}}\right)\right] \end{array}$$ (25)

Eq (25) shows that the higher the speed, the greater the viscosity, the greater the pressure difference, and the greater the viscous friction loss.

Since the author’s research team has previously published a mathematical model of the flow pulsation rate of the SCIGP, it will not be repeated here. In addition, the minimum value of trapped-oil pressure affects the cavitation degree in trapped oil region, and this mathematical model is clear; the maximum value affects the end surface leakage, and this mathematical model can refer to (7).

## 3. SCIGP modeling: Fluid dynamics model

### 3.1 Orthogonal test design

In order to obtain sufficient and effective data with as few test times as possible, and analyze the test results to attain reliable conclusions, the orthogonal method was used to design the test scheme in this study.

#### 3.1.1 Design of factor level table

The factors studied in this paper are the oil film thicknesses between the friction pairs—i.e., the oil film thickness *h*_1_ between the gear end face and the shell, the oil film thickness *h*_2_ between the external gear addendum and the crescent diaphragm, and the oil film thickness *h*_3_ between the internal gear ring addendum and the crescent diaphragm, the oil film thickness *h*_4_ between the inner wall of external gear and the bearing, and the oil film thickness *h*_5_ between the outer wall of internal gear ring and the shell. A total of 5 factors are represented by A, B, C, D, and E respectively. According to the range of oil film thickness, each factor is assumed to be 0.01 mm, 0.02 mm, 0.03 mm and 0.04 mm, respectively. In this way, the factors and levels under investigation are listed in the form shown in Table 1.

**Table 1:** Factor level table.

Factor					
Level	A	B	C	D	E
	oil film thickness on the end face (mm)	oil film thickness on the external gear addendum (mm)	oil film thickness on the internal gear ring addendum (mm)	oil film thickness on the inner wall of external gear (mm)	oil film thickness on the outer wall of internal gear ring (mm)
1	0.01	0.01	0.01	0.01	0.01
2	0.02	0.02	0.02	0.02	0.02
3	0.03	0.03	0.03	0.03	0.03
4	0.04	0.04	0.04	0.04	0.04

#### 3.1.2 Determination of test scheme

According to Table 1, it is necessary to design an equal level orthogonal table with 5 factors and 4 levels. The corresponding standard orthogonal table *L*_16_ (4^5^) is selected. The 16 specific test conditions can be procured, and the corresponding test schemes are shown in Table 2.

**Table 2:** *L*_16_ (4^5^) test scheme.

Test number	Factor				
	A	B	C	D	E
1	1	1	1	1	1
2	2	1	2	2	2
3	3	1	3	3	3
4	4	1	4	4	4
5	2	2	1	3	4
6	1	2	2	4	3
7	4	2	3	1	2
8	3	2	4	2	1
9	3	3	1	4	2
10	4	3	2	3	1
11	1	3	3	2	4
12	2	3	4	1	3
13	4	4	1	2	3
14	3	4	2	1	4
15	2	4	3	4	1
16	1	4	4	3	2

#### 3.1.3 Selection of test objectives

In order to fully analyze the influence of oil film thickness on the performance of the SCIGP, the test targets selected in this study are flow pulsation rate, pressure pulsation rate, trapped oil pressure, volumetric efficiency and total efficiency.

### 3.2 CFD simulation model

#### 3.2.1 Establishment of three-dimensional fluid finite element model

**Fig 8:**
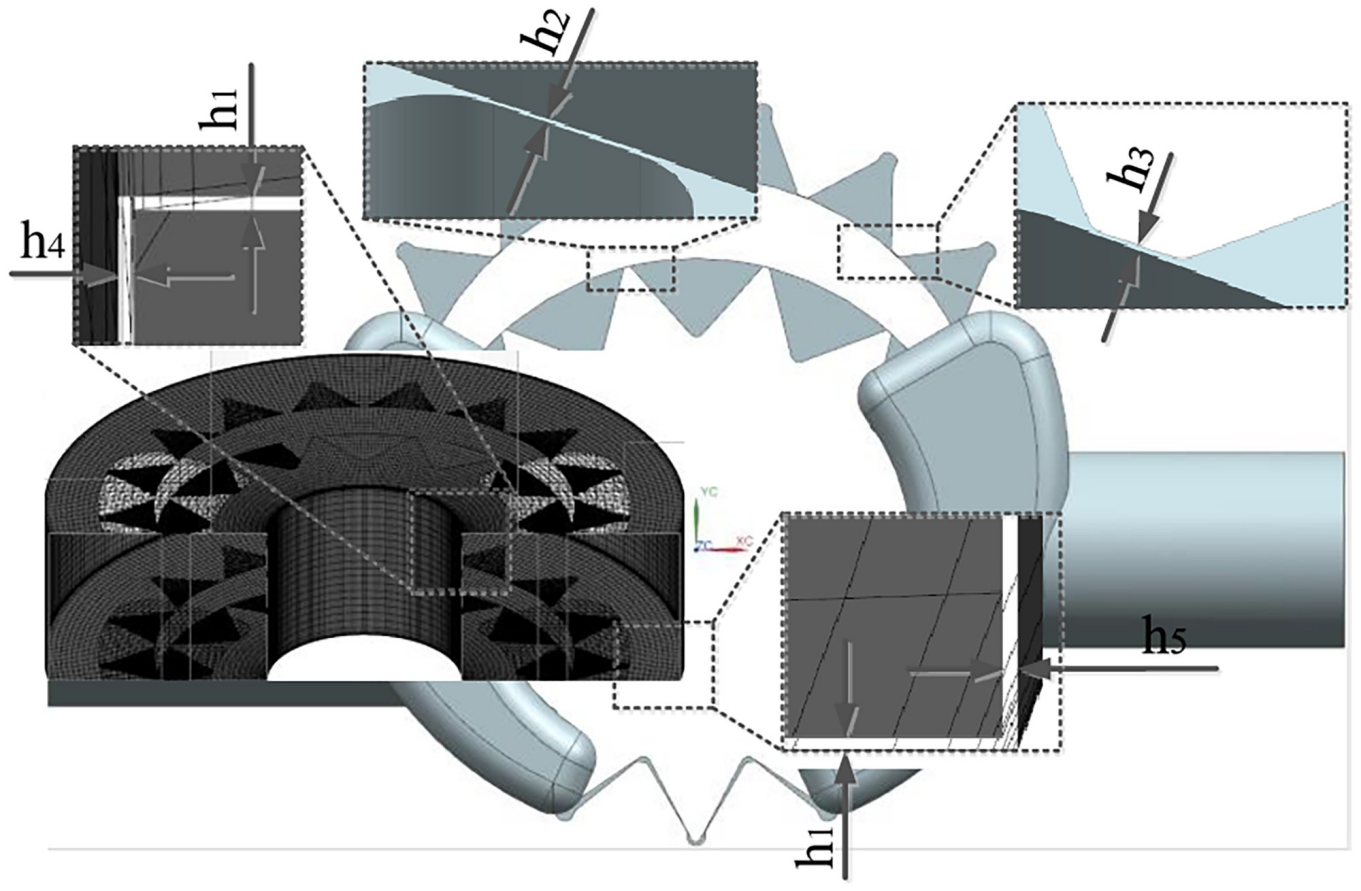
Three-dimensional fluid model considering the oil film thickness.

According to the above analysis results, the three-dimensional fluid models with different addendum oil film thickness are established in the modeling software. Meanwhile, the different oil film thicknesses in the axial direction, the inner wall of external gear and the outer wall of internal ring gear are respectively set in the CFD simulation software. The fluid model is shown in Fig 8.

The grid model in Fig 8 is divided by a combination of Cartesian grid and structured dynamic grid, and the MGI technology is used to set the interface between the dynamic and static fluid domains. The mesh quality is aimed at no surface fragments, and the mesh in the moving area is adaptively refined.

According to the type of gear pump, the main performance parameters of the pump are listed, as shown in Table 3.

**Table 3:** Main performance parameters of gear pump.

Displacement (ml/r)	Maximum speed (r/min)	Outlet pressure (MPa)		Mineral oil (mm^2^/s)
		Rated value	Peak value	Viscosity range
51.1	2600	12.5	16.0	10~100

#### 3.2.2 Setting of simulation parameters

As one of the three most critical stages in flow simulation, the parameters of flow channel model directly affect the speed of simulation process and the accuracy of simulation results. Therefore, setting reasonable simulation parameters has far-reaching significance.

*1) Boundary conditions*. According to the working condition of gear pump (i.e., the inlet and outlet pressures are known), the inlet and outlet boundary conditions are set as pressure inlet and pressure outlet, and the values are 0.1 MPa and 12.5 MPa respectively. The external gear speed is set as 2000 r/min. According to the definition of transmission ratio, the internal gear ring speed is 1529.4 r/min. The inner and outer cylinder surfaces of the inner wall oil film of external gear are set as the rotating surfaces, and the speeds are both 2000 r/min. The inner cylinder surface of the outer wall oil film of internal gear ring is set as the rotating surface, and the speed is 1529.4 r/min.

*2) Flowing medium*. According to the viscosity range and the working conditions of the gear pump oil, 46# mineral oil is selected as the flowing medium. According to the gas condition of the mineral oil [22], the gas content is set as 0.5%. According to the measured data, the maximum oil temperature of general hydraulic system is 60°C under stable working conditions, and the commonly used value of 50°C is used in this study. This paper assumes that the working conditions (i.e., pressure, oil temperature and gas content) do not change over time, so that the three major characteristics of the oil (i.e., mass characteristics, stiffness characteristics, and damping characteristics) remain basically unchanged. The physical properties of the oil under this working condition are listed in Table 4.

**Table 4:** 46# mineral oil media properties.

Gas content (%)	Oil temperature (°C)	System pressure (MPa)	Density (kg/m3)	Dynamic viscosity (Pa.s)	Bulk modulus (MPa)
0.5	50	12.5	849.7	0.031	1670.114

*3) Turbulence model*. Due to the high speed of gear pair and the effect of high pressure difference, the oil velocity in the meshing region is very high, and the streamlines are intertwined. In order to simulate this complex flow with transient flow and curved streamlines, the RNG k − ε turbulence model is used in the rotor region to deal with the streamlines with high strain rate and large bending degree in the internal channel, and the near wall is treated more reasonably. The standard k − ε turbulence model is used in other regions.

*4) Number of time steps*. Under the premise of not losing the calculation accuracy and speeding up the calculation speed, the variable time step is adopted, and the maximum number of iterations in each time step is set to 100, and the number of time steps required for the external gear to rotate one tooth is set to 30. Since the number of external gear teeth is 13, that is, 390 steps are a cycle. According to the external gear speed (2000 r/min), the motion period is 0.03 s.

*5) Cavitation model*. The equilibrium dissolved gas model is selected as the cavitation model. The model uses gas migration to determine the mass fraction of non-condensable gas (NCG) dissolved in liquid, and assumes that the dissolved gas is in equilibrium. The gas is dissolved according to the balance between the local pressure and the dissolved gas reference pressure.

## 4. Calculation results and analysis

### 4.1 Independence verification of grid and convergence criteria

#### 4.1.1 Grid independence verification

It is verified that the increase of grid nodes and grid quality have little effect on the accuracy of the calculation results. The grid model used in this study has good quality, so it is only necessary to verify the influence of the nodes number on the calculation results. The specific results are shown in Table 5.

**Table 5:** Simulation results corresponding to different nodes numbers.

Grids number	Nodes number	Average outlet flow (L/min)	Deviation rate (%)
454430	792420	94.552	2.61
567169	1045980	95.736	1.39
603585	1035390	96.975	0.11
735362	1312560	97.067	0.02
793642	1483569	97.082	0.01

Table 5 shows that when the nodes number increases to 1.03539 million, the deviation rate decreases to within 0.1%. Therefore, in order to speed up the calculation speed, the grid model with 1.03539 million nodes is selected for numerical simulation.

#### 4.1.2 Independence verification of convergence criteria

The calculation result basically does not change after the convergence criterion is reduced to a certain value. This study calculated the outlet flow when the convergence criteria were 0.1, 0.06, 0.02, 0.01 and 0.001, respectively. The specific results are shown in Table 6.

**Table 6:** Simulation results corresponding to different convergence criterion.

Convergence standard	Average outlet flow (L/min)	Deviation rate (%)
0.1	96.325	0.47
0.06	96.638	0.15
0.02	96.706	0.08
0.01	96.752	0.03
0.001	96.783	0.01

In Table 6, the convergence criterion has very little influence on the calculation results. In order to further improve the calculation accuracy, the convergence criteria is set to 0.01.

### 4.2 Internal flow field distribution during the meshing process of gear pair

**Fig 9:**
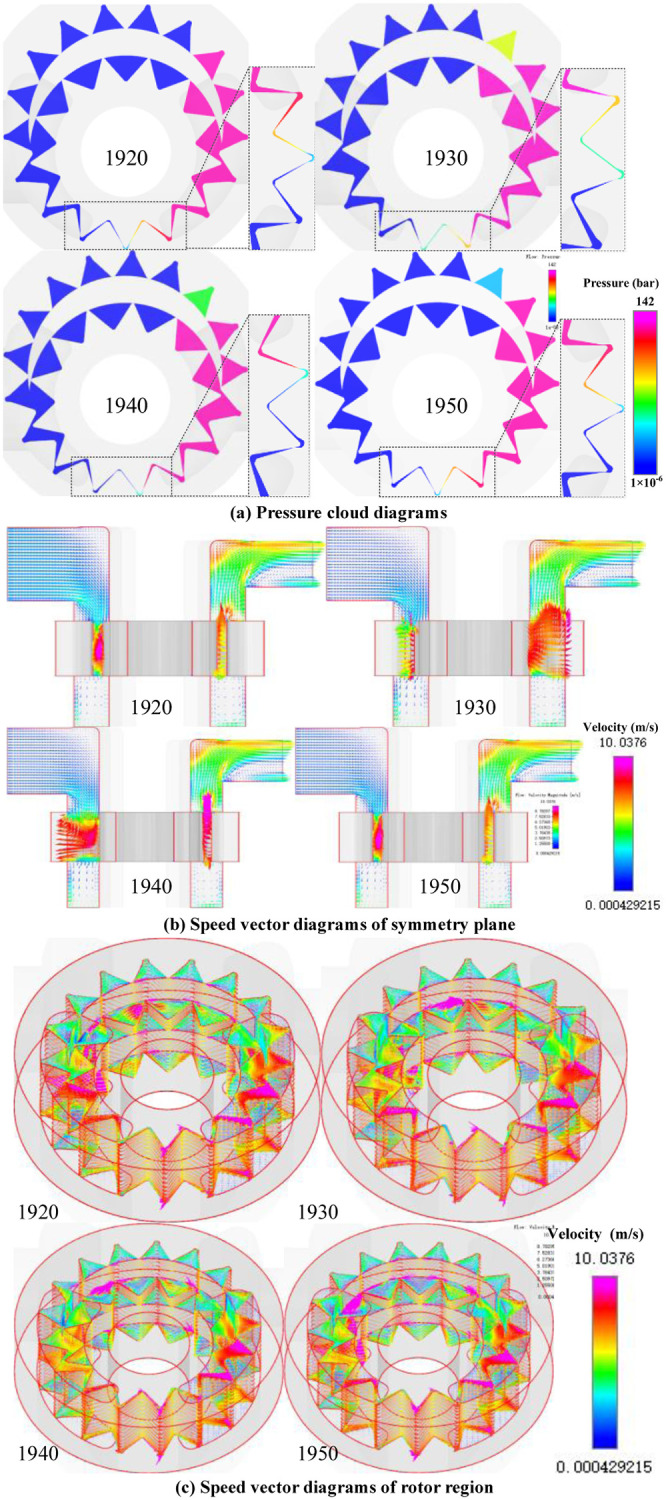
Distribution diagram of internal flow field. (a) Pressure cloud diagrams. (b) Speed vector diagrams of symmetry plane. (c) Speed vector diagrams of rotor region.

Since the internal flow field distributions under different test conditions are similar, test 1 is taken as an example to obtain the pressure cloud diagrams, and the speed vector diagrams of symmetry plane and rotor region during the process of external gear from entering meshing (time step 1920) to exiting meshing (time step 1950), as shown in Fig 9.

From the pressure cloud diagrams in Fig 9, it can be seen that the oil film between the tooth flanks, the axial and radial oil film completely isolates the oil-suction cavity and the oil-discharge cavity, and the pressure distribution between the high and low pressure regions is uniformly transitioned. The change of trapped oil volume first decreases and then increases in the process of gear pair from entering meshing to exiting meshing, and the pressure changes inversely.

The velocity vector diagrams show that under the meshing transmission of gear pair, the oil in the oil-suction cavity is continuously transported to the oil-discharge cavity through the inter-tooth cavity. The particle flow in the oil-suction and oil-discharge regions is obviously stratified, and the particle flow in the rotor region is in a complex and irregular state. The particle velocity in the addendum and tooth flank clearances is the largest, reaching 10 m/s, and the flow velocity in the rest is relatively low.

### 4.3 Comparison of flow pulsation under different test conditions

**Fig 10:**
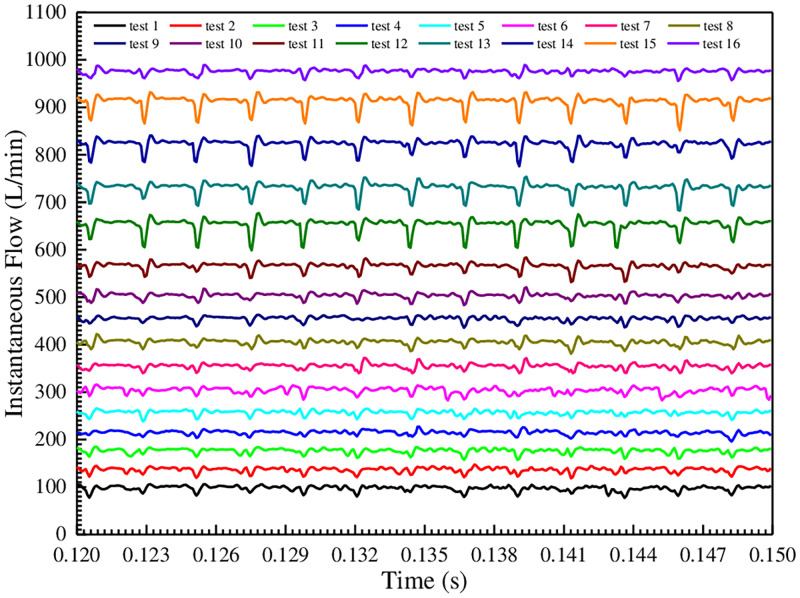
Comparison of instantaneous flow curves under different test conditions.

The flow pulsation rate is a key indicator to evaluate the instantaneous flow quality of hydraulic pumps. The output instantaneous flow of gear pump is always fluctuating, which is caused by the periodic change of meshing point position, the uneven internal leakage, the oil compressibility and the trapped oil phenomenon, etc. Therefore, the outlet instantaneous flow curves of gear pump under different test conditions are evaluated and compared, as shown in Fig 10.

**Fig 11:**
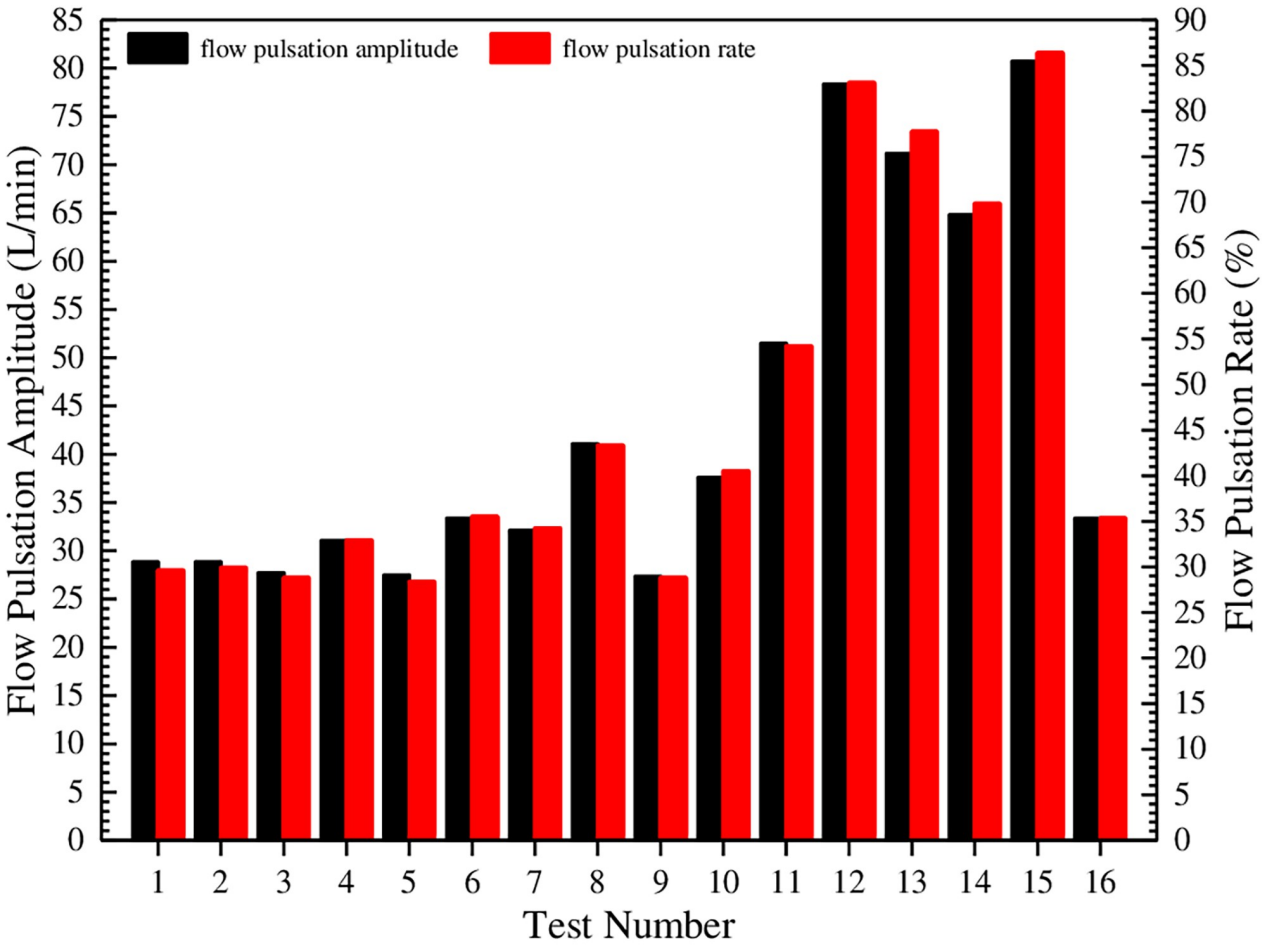
Comparison of flow pulsation amplitude and pulsation rate under different test conditions.

Fig 10 shows that under different test conditions, the instantaneous flow curves in each cycle show continuous periodic changes, and there are 13 fluctuation pulsations. The reason is that the oil at the outlet of the pump is transmitted through the meshing transmission of the gear pair, and the gear pair meshes 13 times in a movement cycle. In addition, the instantaneous flow curves under different test conditions are not the same, which is the result of the comprehensive influence due to the inhomogeneity of internal leakage flow, the difference of compression flow and trapped oil flow, etc. It can be seen from Reference [11] that the minimum value of instantaneous flow corresponds to the position of gear pair exiting meshing. Then, the pulsation amplitude and the corresponding pulsation rate of instantaneous flow in Fig 10 are procured and plotted as a histogram, as shown in Fig 11.

Fig 11 shows that the flow pulsation amplitude and the pulsation rate are large as a whole, especially in tests 12, 13, 14 and 15. Among them, the pulsation amplitude and the pulsation rate of test 15 are the largest, which are 80.702 L/min and 86.384%, respectively. Test 9 is the smallest. In order to further estimate the influence degree of test factors on the flow pulsation rate and determine the optimal level combination, an orthogonal test analysis table for the flow pulsation rate is established, as shown in Table 7.

**Table 7:** Orthogonal test analysis table of flow pulsation rate.

Test number	Factor					Test result
	A	B	C	D	E	Flow pulsation rate (%)
1	1	1	1	1	1	29.618
2	2	1	2	2	2	29.902
3	3	1	3	3	3	28.798
4	4	1	4	4	4	32.897
5	2	2	1	3	4	28.353
6	1	2	2	4	3	35.533
7	4	2	3	1	2	34.221
8	3	2	4	2	1	43.326
9	3	3	1	4	2	28.825
10	4	3	2	3	1	40.498
11	1	3	3	2	4	54.197
12	2	3	4	1	3	83.121
13	4	4	1	2	3	77.745
14	3	4	2	1	4	69.816
15	2	4	3	4	1	86.384
16	1	4	4	3	2	35.325
k1	38.668	30.304	41.135	54.194	49.957	
k2	56.940	35.358	43.937	51.293	32.068	
k2	42.691	51.660	50.900	33.244	56.299	
k4	46.341	67.318	48.667	45.910	46.316	
R	18.272	37.014	9.7648	20.951	24.231	

According to the range-method, the primary and secondary relationship of the factors affecting the flow pulsation rate in Table 7 is: B>E>D>A>C, that is, the oil film thickness on the external gear addendum is the largest factor, and the other factors are the oil film thickness on the outer wall of internal gear ring, the oil film thickness on the inner wall of external gear and the oil film thickness on the end face in sequence. The influence of the oil film thickness on the internal gear ring addendum can be ignored. Therefore, in order to procure a lower flow pulsation rate, the smaller the oil film thickness on the external gear addendum, the better. Furthermore, Table 7 shows that the level combination with the lowest pulsation rate is A_1_B_1_C_1_D_3_E_2_, that is, the oil film thicknesses on the end face, on the external gear addendum, and on the internal gear ring addendum are all 0.01 mm, and the oil film thicknesses on the inner wall of external gear and on the outer wall of internal gear ring are 0.03 mm and 0.02 mm, respectively. It has been verified that the flow pulsation rate under this combination is 26.835%.

### 4.4 Comparison of pressure pulsation under different test conditions

**Fig 12:**
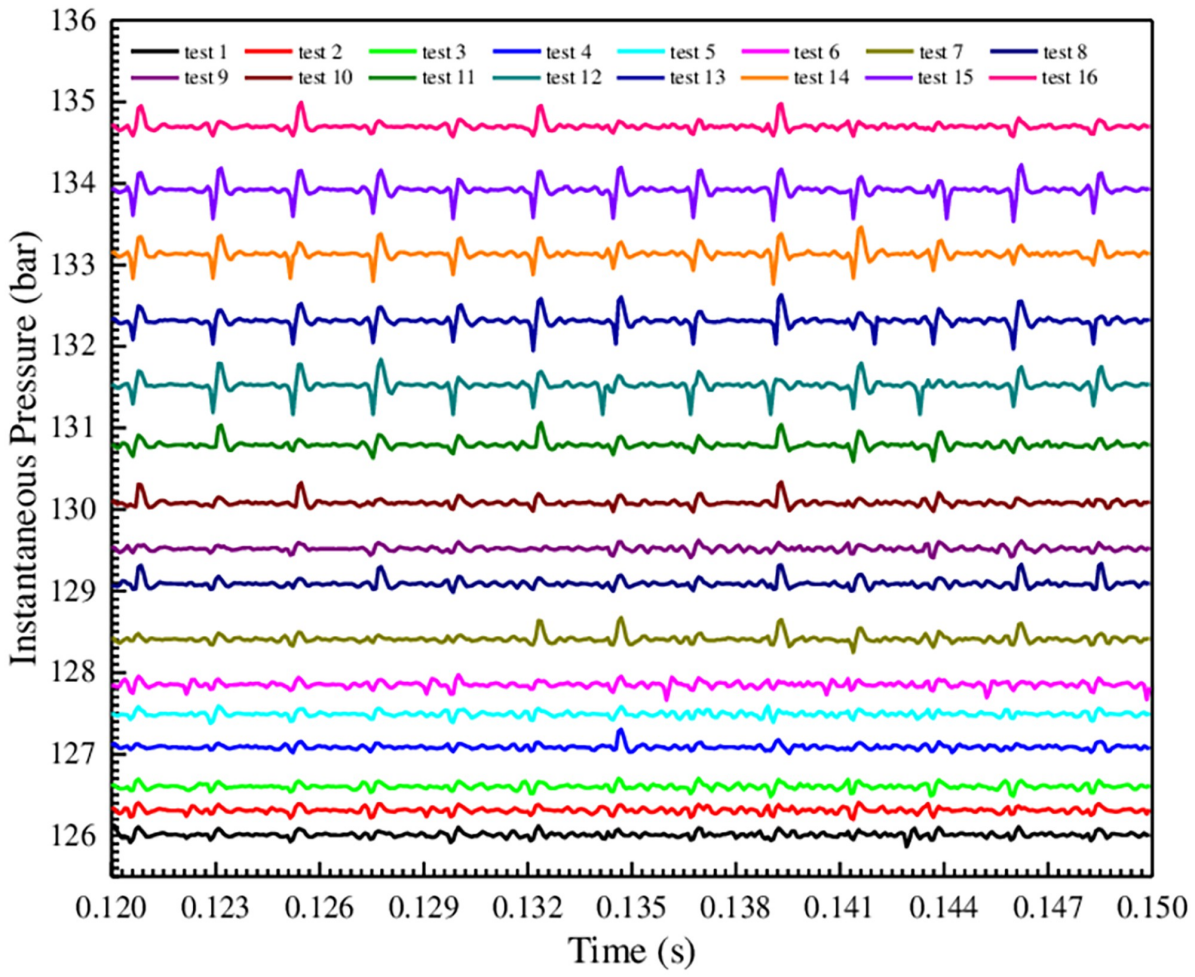
Comparison of instantaneous outlet pressure curves under different test conditions.

The pressure pulsation is inspired by the pulsating flow. The pulsating high-frequency peak pressure is not only the main factor for the plastic deformation of the weak parts in the hydraulic components, but also generates impact noise, etc. Therefore, the instantaneous outlet pressure curves of gear pump under different test conditions are procured and compared, as shown in Fig 12.

**Fig 13:**
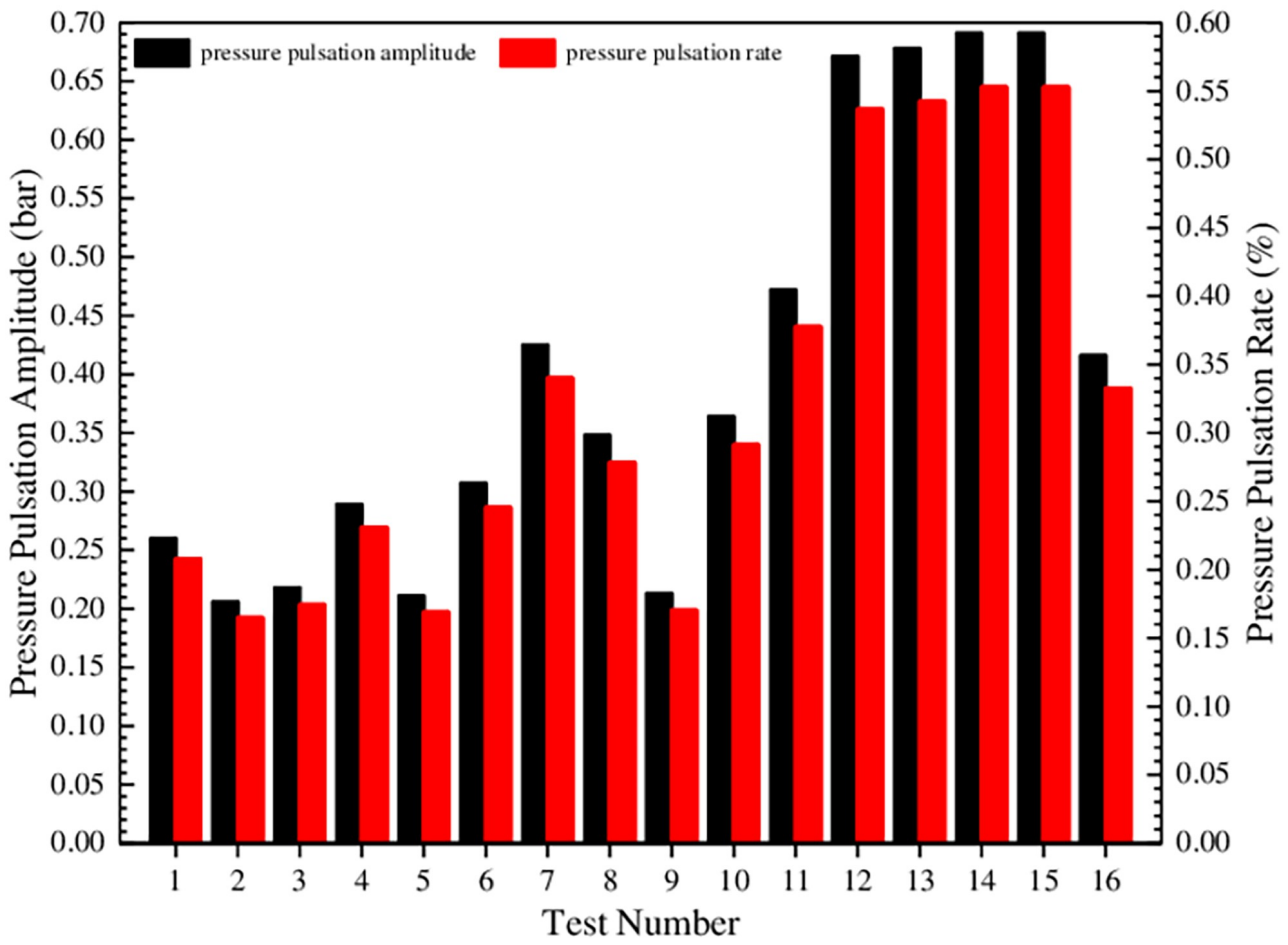
Comparison of pressure pulsation amplitude and pulsation rate under different test conditions.

Fig 12 shows that under different test conditions, the trend of the pulsating pressure and flow curves is completely consistent, and the pressure pulsation is positively correlated with the flow pulsation. Then the pulsation amplitude and the corresponding pulsateon rate of the instantaneous pressure in Fig 12 are procured and plotted as a histogram, as shown in Fig 13.

Fig 13 shows that the pressure pulsation amplitude and pulsation rate are both small under the entire test conditions, and the maximum values are 0.0691 MPa and 0.5528%, respectively. Due to the same average pressure, the high pulsation amplitude under different test conditions is positively correlated with the high pulsation rate. Among them, the pulsation rate of test 9 is the smallest, and test 15 is the largest. In order to further estimate the influence degree of test factors on pressure pulsation rate and determine the optimal level combination, the orthogonal test analysis table of the pressure pulsation rate is established, as shown in Table 8.

**Table 8:** Orthogonal test analysis table of pressure pulsation.

Test number	Factor					Test result
	A	B	C	D	E	Pressure pulsation rate (%)
1	1	1	1	1	1	0.208
2	2	1	2	2	2	0.165
3	3	1	3	3	3	0.174
4	4	1	4	4	4	0.231
5	2	2	1	3	4	0.169
6	1	2	2	4	3	0.246
7	4	2	3	1	2	0.340
8	3	2	4	2	1	0.278
9	3	3	1	4	2	0.170
10	4	3	2	3	1	0.291
11	1	3	3	2	4	0.378
12	2	3	4	1	3	0.537
13	4	4	1	2	3	0.542
14	3	4	2	1	4	0.553
15	2	4	3	4	1	0.553
16	1	4	4	3	2	0.333
k1	0.291	0.195	0.272	0.409	0.333	
k2	0.356	0.258	0.314	0.341	0.252	
k2	0.294	0.344	0.361	0.242	0.375	
k4	0.351	0.495	0.345	0.300	0.333	
R	0.065	0.301	0.089	0.168	0.123	

It can be seen from Table 8 that the primary and secondary relationship of the factors affecting the pressure pulsation rate is: B>D>E>C>A, that is, the oil film thickness on the external gear addendum is the largest factor, and the other factors are the oil film thickness on the inner wall of external gear and the oil film thickness on the outer wall of internal gear ring in sequence. The influence of the oil film thickness on the internal gear ring addendum and the oil film thickness on the end face can be ignored. Therefore, in order to procure a lower pressure pulsation rate, the smaller the oil film thickness on the external gear addendum, the better. Furthermore, Table 8 shows that the level combination with the lowest pulsation rate is also A_1_B_1_C_1_D_3_E_2_, that is, the oil film thicknesses on the end face, on the external gear addendum, and on the internal gear ring addendum are all 0.01 mm, and the oil film thicknesses on the inner wall of external gear and on the outer wall of internal gear ring are 0.03 mm and 0.02 mm, respectively. It has been verified that the pressure pulsation rate under this combination is 0.158%.

### 4.5 Comparison of trapped oil pressure under different test conditions

**Fig 14:**
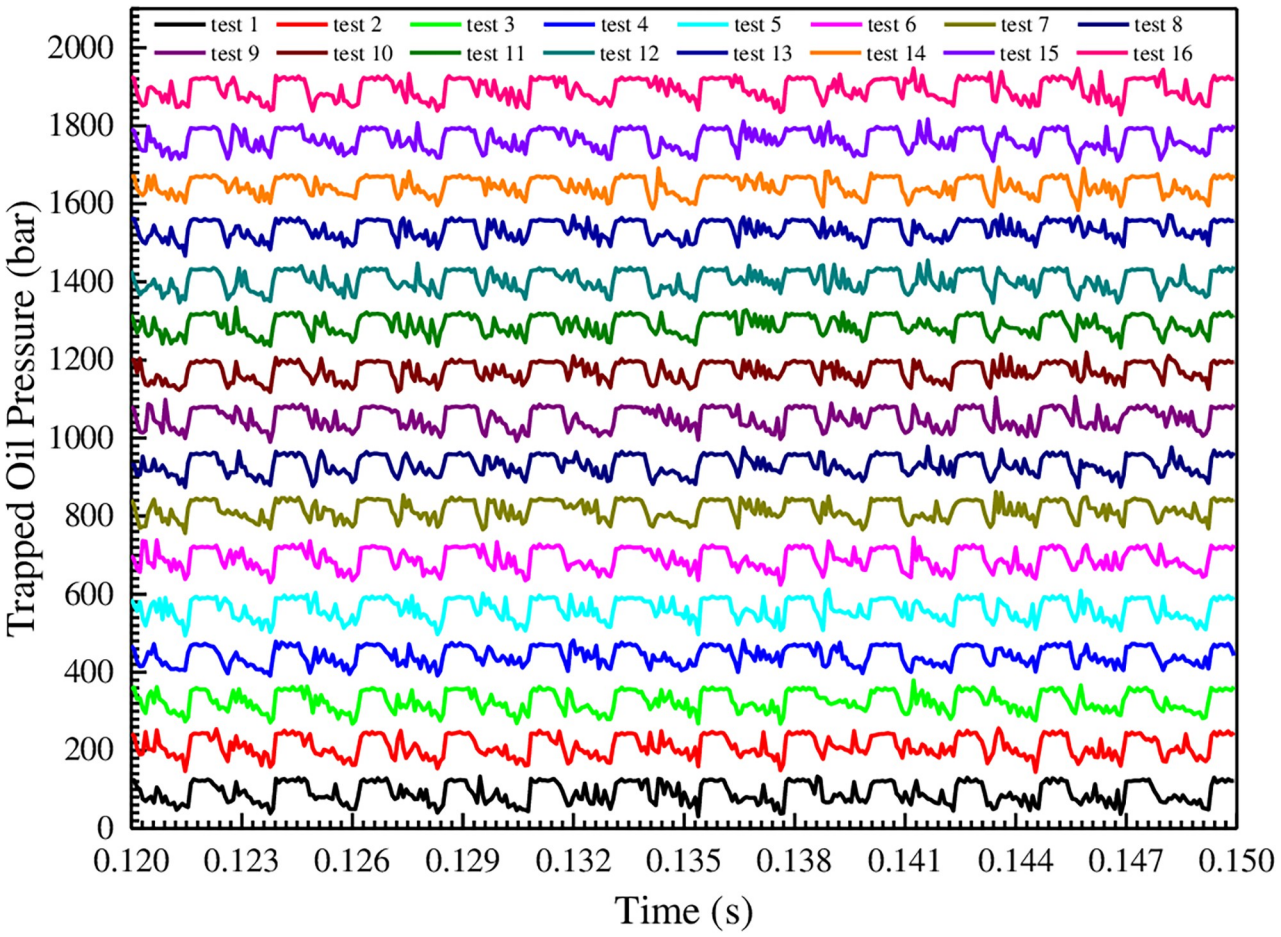
Comparison of trapped oil pressure curves under different test conditions.

The level of trapped oil pressure not only causes the gear to withstand periodic pressure impact, but also generates local vacuum and cavitation, and even cavitation erosion, as well as vibration and noise, etc. Therefore, the trapped oil pressure curves of gear pump under different test conditions are procured and compared, as shown in Fig 14.

**Fig 15:**
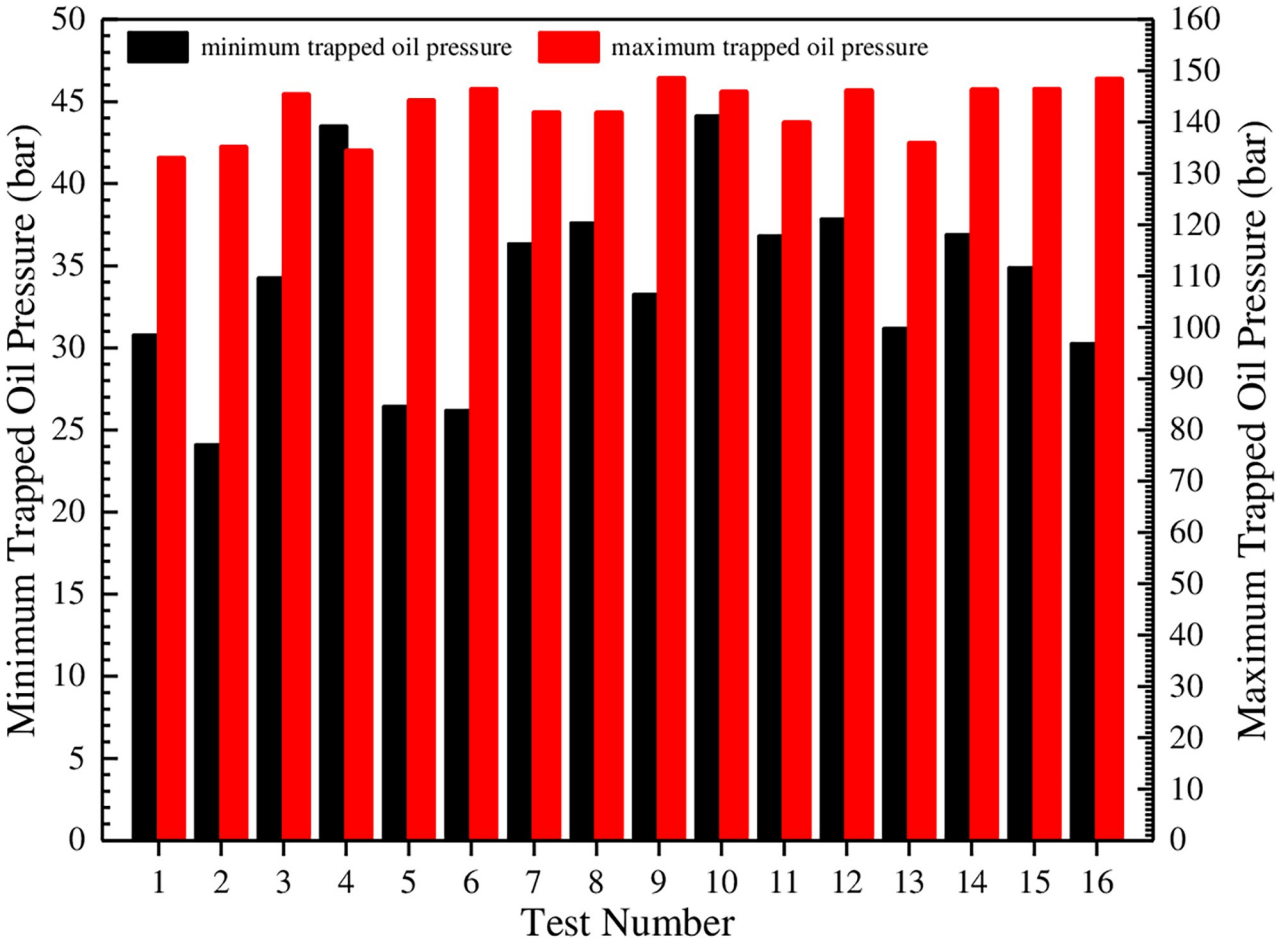
Comparison of the maximum and the minimum of trapped oil pressure under different test conditions.

Fig 14 shows that the trapped oil pressure curves are ladder-shaped distribution, that is, in the process of gear meshing, the higher pressure value maintains about 1/5 of the time, and then begins to decrease. Under different test conditions, the trapped oil pressure curves in each cycle show continuous periodic changes, and there are 13 fluctuation pulsations. Then the maximum and the minimum of trapped oil pressure in Fig 14 are procured and plotted as a histogram, as shown in Fig 15.

Fig 15 shows that the minimum value of trapped oil pressure varies greatly under different test conditions, and the minimum of test 2 is the smallest, and it is 2.4 MPa. This value is much higher than the air separation pressure of oil with 10% air content at 100°C [23]. Therefore, for the gear pumps with the tooth profile curves, there will be no cavitation in the oil trapped region. In addition, the maximum value of trapped oil pressure is relatively close. The maximum of test 9 is the largest, and it is 14.86 MPa, and this value will augment the leakage flow and aggravate the flow pulsation rate.

In order to further estimate the influence degree of test factors on the trapped oil pressure and determine the optimal level combination, the orthogonal test analysis tables of the minimum and the maximum of trapped oil pressure are established, as shown in Tables 9 and 10.

**Table 9:** Orthogonal test analysis table of the minimum trapped oil pressure.

Test number	Factor					Test result
	A	B	C	D	E	Minimum trapped oil pressure (%)
1	1	1	1	1	1	30.746
2	2	1	2	2	2	24.068
3	3	1	3	3	3	34.239
4	4	1	4	4	4	43.488
5	2	2	1	3	4	26.400
6	1	2	2	4	3	26.165
7	4	2	3	1	2	36.316
8	3	2	4	2	1	37.585
9	3	3	1	4	2	33.222
10	4	3	2	3	1	44.095
11	1	3	3	2	4	36.784
12	2	3	4	1	3	37.825
13	4	4	1	2	3	31.160
14	3	4	2	1	4	36.879
15	2	4	3	4	1	34.855
16	1	4	4	3	2	30.233
k1	30.982	33.135	30.382	35.441	36.820	
k2	30.787	31.616	32.802	32.400	30.960	
k2	35.481	37.981	35.549	33.742	32.347	
k4	38.765	33.282	37.283	34.433	35.888	
R	7.978	6.365	6.901	3.042	5.861	

**Table 10:** Orthogonal test analysis table of the maximum trapped oil pressure.

Test number	Factor					Test result
	A	B	C	D	E	Maximum trapped oil pressure (%)
1	1	1	1	1	1	132.97
2	2	1	2	2	2	135.13
3	3	1	3	3	3	145.36
4	4	1	4	4	4	134.39
5	2	2	1	3	4	144.24
6	1	2	2	4	3	146.41
7	4	2	3	1	2	141.90
8	3	2	4	2	1	141.83
9	3	3	1	4	2	148.56
10	4	3	2	3	1	145.90
11	1	3	3	2	4	139.95
12	2	3	4	1	3	146.16
13	4	4	1	2	3	135.94
14	3	4	2	1	4	146.31
15	2	4	3	4	1	146.40
16	1	4	4	3	2	148.41
k1	141.93	136.96	140.43	141.84	141.77	
k2	142.98	143.59	143.44	138.21	143.50	
k2	145.52	145.14	143.40	145.98	143.47	
k4	138.04	144.26	142.70	143.94	141.22	
R	7.472	8.178	3.009	7.765	2.280	

Table 9 shows that the primary and secondary relationship of the factors affecting the minimum trapped oil pressure is: A>C>B>E>D, that is, the oil film thickness on the end face is the largest factor, and the other factors are the oil film thickness on the internal gear ring addendum, the oil film thickness on the external gear addendum, the oil film thickness on the outer wall of internal gear ring and the oil film thickness on the inner wall of external gear in sequence. Therefore, in order to procure a higher minimum trapped oil pressure, the oil film thicknesses on the end face and on the internal gear ring addendum should be increased as much as possible. Furthermore, Table 9 shows that the level combination with the highest minimum is A_4_B_3_C_4_D_1_E_1_, that is, the oil film thicknesses on the end face and on the internal gear ring addendum are both 0.04 mm, the oil film thickness on the external gear addendum is 0.03 mm, and the oil film thicknesses on the inner wall of external gear and on the outer wall of internal gear ring are both 0.01 mm. It has been verified that the minimum trapped oil pressure under this combination is 4.5326 MPa.

Table 10 shows that the primary and secondary relationship of the factors affecting the maximum trapped oil pressure is: B>D>A>C>E, that is, the oil film thickness on the external gear addendum is the largest factor, and the other factors are the oil film thickness on the inner wall of external gear and the oil film thickness on the end face in sequence. The influence of the oil film thickness on the internal gear ring addendum and on the outer wall of internal gear ring can be ignored. Therefore, in order to procure a lower maximum trapped oil pressure, the greater the oil film thickness on the external gear addendum, the better. Further- more, Table 10 shows that the level combination with the lowest maximum is A_4_B_1_C_1_D_2_E_4_, that is, the oil film thicknesses on the end face and on the outer wall of internal gear ring are both 0.04 mm, the oil film thicknesses on the external gear addendum and on the internal gear ring addendum are both 0.01 mm, and the oil film thickness on the inner wall of external gear is 0.02 mm. It has been verified that the maximum trapped oil pressure under this combination is 13.1267 MPa.

### 4.6 Comparison of volumetric efficiency and total efficiency under different test conditions

**Fig 16:**
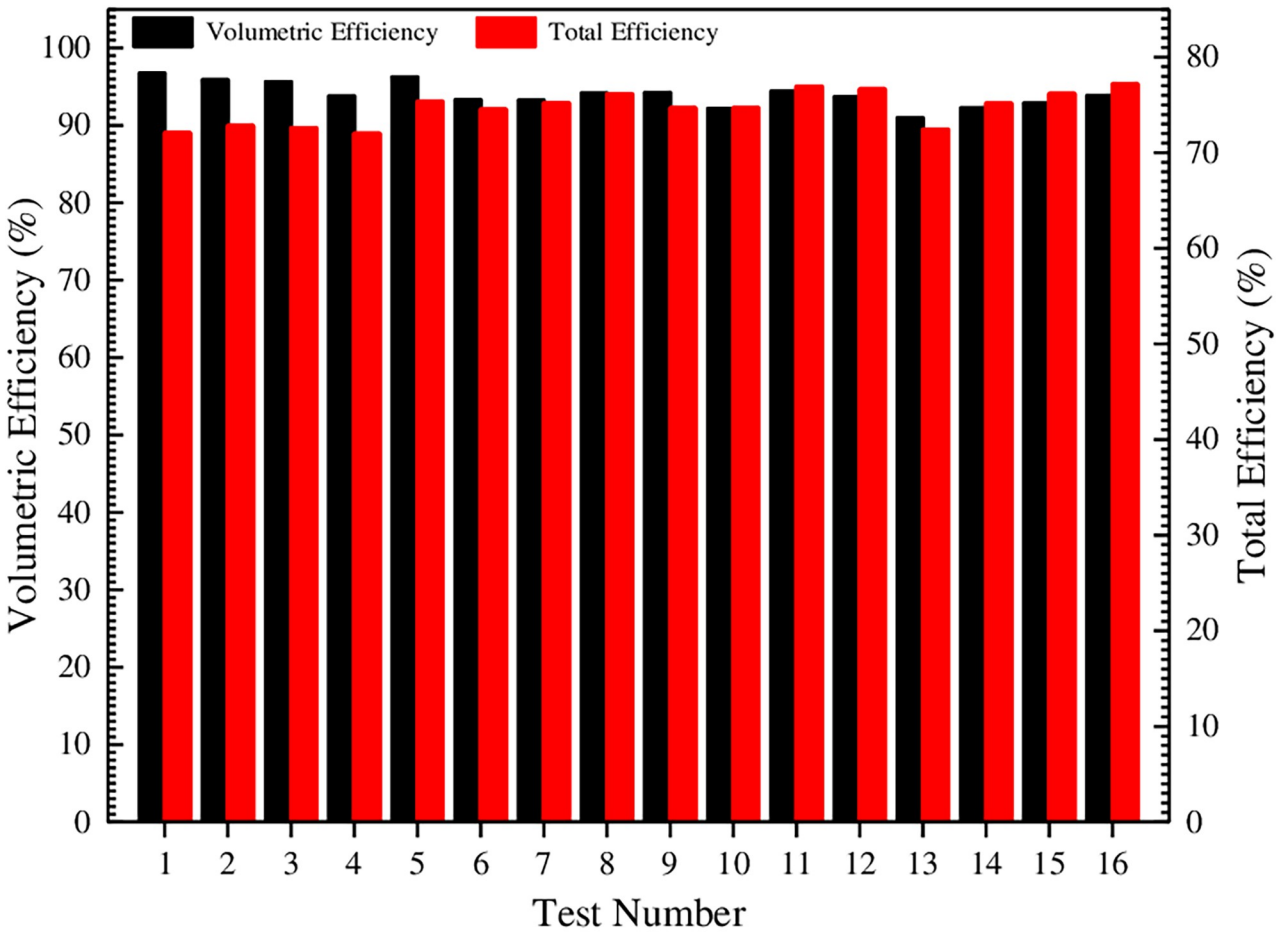
Comparison of volumetric efficiency and total efficiency under different test conditions.

In the above part of this study, the mathematical models of internal leakage and viscous friction loss under different clearances in the SCIGP are derived in detail, which lays a theoretical foundation for the analysis of numerical calculation and experimental test results. Then the volumetric efficiency and total efficiency under different test conditions are procured and plotted as a histogram, as shown in Fig 16.

In Fig 16, the volumetric efficiency under different test conditions is between 90%-97%, and the total efficiency is between 72%-77.5%. Due to the difference in the degree of viscous friction loss, the volumetric efficiency is not positively correlated with the total efficiency. The volumetric efficiency of test 1 is the largest, and the total efficiency of test 16 is the highest. In order to further estimate the influence degree of test factors on the volumetric efficiency and the total efficiency, and determine the optimal level combination, the orthogonal test analysis tables for volumetric efficiency and total efficiency are established respectively, as shown in Tables 11 and 12.

**Table 11:** Orthogonal test analysis table of volumetric efficiency.

Test number	Factor					Test result
	A	B	C	D	E	Volumetric efficiency (%)
1	1	1	1	1	1	96.736
2	2	1	2	2	2	95.874
3	3	1	3	3	3	95.612
4	4	1	4	4	4	93.774
5	2	2	1	3	4	96.243
6	1	2	2	4	3	93.272
7	4	2	3	1	2	93.230
8	3	2	4	2	1	94.162
9	3	3	1	4	2	94.234
10	4	3	2	3	1	92.172
11	1	3	3	2	4	94.408
12	2	3	4	1	3	93.680
13	4	4	1	2	3	90.947
14	3	4	2	1	4	92.233
15	2	4	3	4	1	92.865
16	1	4	4	3	2	93.831
k1	94.562	95.499	94.540	93.970	93.984	
k2	94.665	94.227	93.388	93.848	94.292	
k2	94.060	93.624	94.029	94.465	93.377	
k4	92.531	92.469	93.862	93.536	94.165	
R	2.1348	3.0296	1.1520	0.9285	0.9148	

**Table 12:** Orthogonal test analysis table of total efficiency.

Test number	Factor					Test result
	A	B	C	D	E	Total efficiency (%)
1	1	1	1	1	1	72.075
2	2	1	2	2	2	72.810
3	3	1	3	3	3	72.600
4	4	1	4	4	4	72.006
5	2	2	1	3	4	75.341
6	1	2	2	4	3	74.546
7	4	2	3	1	2	75.175
8	3	2	4	2	1	76.140
9	3	3	1	4	2	74.679
10	4	3	2	3	1	74.690
11	1	3	3	2	4	76.927
12	2	3	4	1	3	76.647
13	4	4	1	2	3	72.443
14	3	4	2	1	4	75.149
15	2	4	3	4	1	76.178
16	1	4	4	3	2	77.175
k1	75.181	72.373	73.635	74.761	74.771	
k2	75.244	75.301	74.299	74.580	74.960	
k2	74.642	75.736	75.220	74.952	74.059	
k4	73.579	75.236	75.492	74.352	74.856	
R	1.6657	3.3628	1.8573	0.5993	0.9005	

Table 11 shows that the primary and secondary relationship of the factors affecting the volumetric efficiency is: B>A>C>D>E, that is, the oil film thickness on the external gear addendum is the largest factor, and the other factors are the oil film thickness on the end face and the oil film thickness on the internal gear ring addendum in sequence. The influence of the oil film thicknesses on the inner wall of external gear and on the outer wall of internal gear ring is small. Therefore, in order to procure a higher volumetric efficiency, the oil film thicknesses on the external gear addendum, on the internal gear ring addendum and on the end face is reduced as much as possible. Furthermore, Table 11 shows that the level combination with the highest volumetric efficiency is A_2_B_1_C_1_D_3_E_2_, that is, the oil film thicknesses on the end face and on the outer wall of internal gear ring are both 0.02 mm, the oil film thicknesses on the external gear addendum and on the internal gear ring addendum are both 0.01 mm, the oil film thickness on the inner wall of external gear is 0.03 mm. It has been verified that the volumetric efficiency under this combination is 97.568%.

Table 12 shows that the primary and secondary relation- ship of the factors affecting the total efficiency is: B>C>A>E>D, that is, the oil film thickness on the external gear addendum is the largest factor, and the other factors are the oil film thickness on the internal gear ring addendum and the oil film thickness on the end face in sequence. The oil film thickness on the outer wall of internal gear ring and the oil film thickness on the inner wall of external gear have little influence. Therefore, in order to procure a higher total efficiency, the oil film thicknesses on the external gear addendum and on the internal gear ring addendum should be appropriately increased, and the oil film thickness on the end face should be minimized. Furthermore, Table 12 shows that the level combination with the highest total efficiency is A_2_B_3_C_4_D_3_E_2_, that is, the oil film thicknesses on the end face and on the outer wall of internal gear ring are both 0.02 mm, the oil film thicknesses on the external gear addendum and on the inner wall of external gear are both 0.03 mm, and the oil film thickness on the internal gear ring addendum is 0.04 mm. It has been verified that the total efficiency under this combination is 77.269%.

### 4.7 Determination of optimum clearance

In summary, the pressure pulsation rates under different working clearance combinations are very small, and there is no cavitation phenomenon and the impact pressure that causes plastic deformation of weak parts in the oil trapped region. Therefore, the consideration for the five test objectives focuses on the flow pulsation rate, the volumetric efficiency and the total efficiency. From the above orthogonal test results, it is not difficult to conclude that the oil film thickness on the external gear addendum is the most important factor affecting these three objectives, followed by the oil film thickness on the end face and the oil film thickness on the internal gear ring addendum. The optimal clearances of friction pairs in the SCIGP can be procured by comprehensively considering the influence of level on each target. That is, the oil film thicknesses on the end face, on the internal gear ring addendum and on the inner wall of external gear are 0.01 mm, 0.04 mm and 0.03 mm respectively, and the oil film thicknesses on the external gear addendum and on the outer wall of internal gear ring are both 0.02 mm.

Then, the simulation model corresponding to the optimal clearances is established and simulated to procure the corresponding simulation values, as shown in Table 13.

**Table 13:** Simulation values corresponding to the optimal clearances.

	Flow pulsation rate (%)	Leakage flow (L/min)	Viscous friction loss (KW)
Simulation result	27.85	4.12	5.29

According to the conclusion of the author’s research team [11], when the outlet pressure increases to the rated pressure, the flow pulsation rate increases from 8.86% to 27.85%. The reason is that the uneven internal leakage is the main factors inducing the surge of pulsation rate. In addition, the theoretical leakage and the friction loss calculated by the mathematical models of internal leakage (13) and viscous friction loss (25) are 0.8884 L/min and 1.7321 KW, respectively. Since the mathematical model is simplified without considering the change of oil flow characteristics, the theoretical value is lower than the simulation value. In order to verify the accuracy of the simulation results, comparative experiments are carried out.

### 4.8 Experimental results and analysis

**Fig 17:**
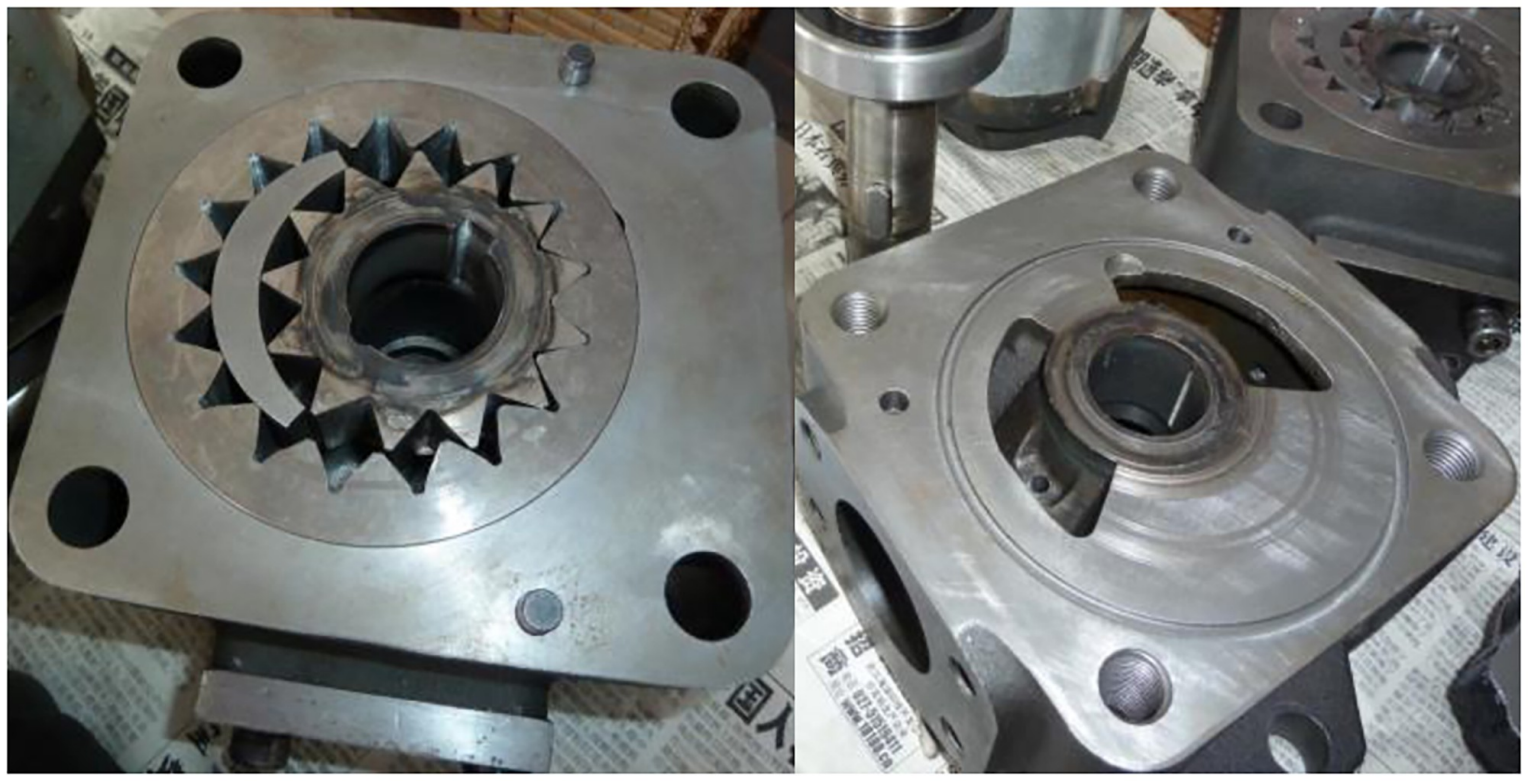
Prototype corresponding to the optimal clearance.

The optimal values of main factors (i.e., oil film thicknesses on the external gear addendum, on the end face and on the internal gear ring addendum) affecting the flow pulsation rate, volumetric efficiency and total efficiency are accurately designed, and the corresponding prototype is manufactured, as shown in Fig 17.

**Fig 18:**
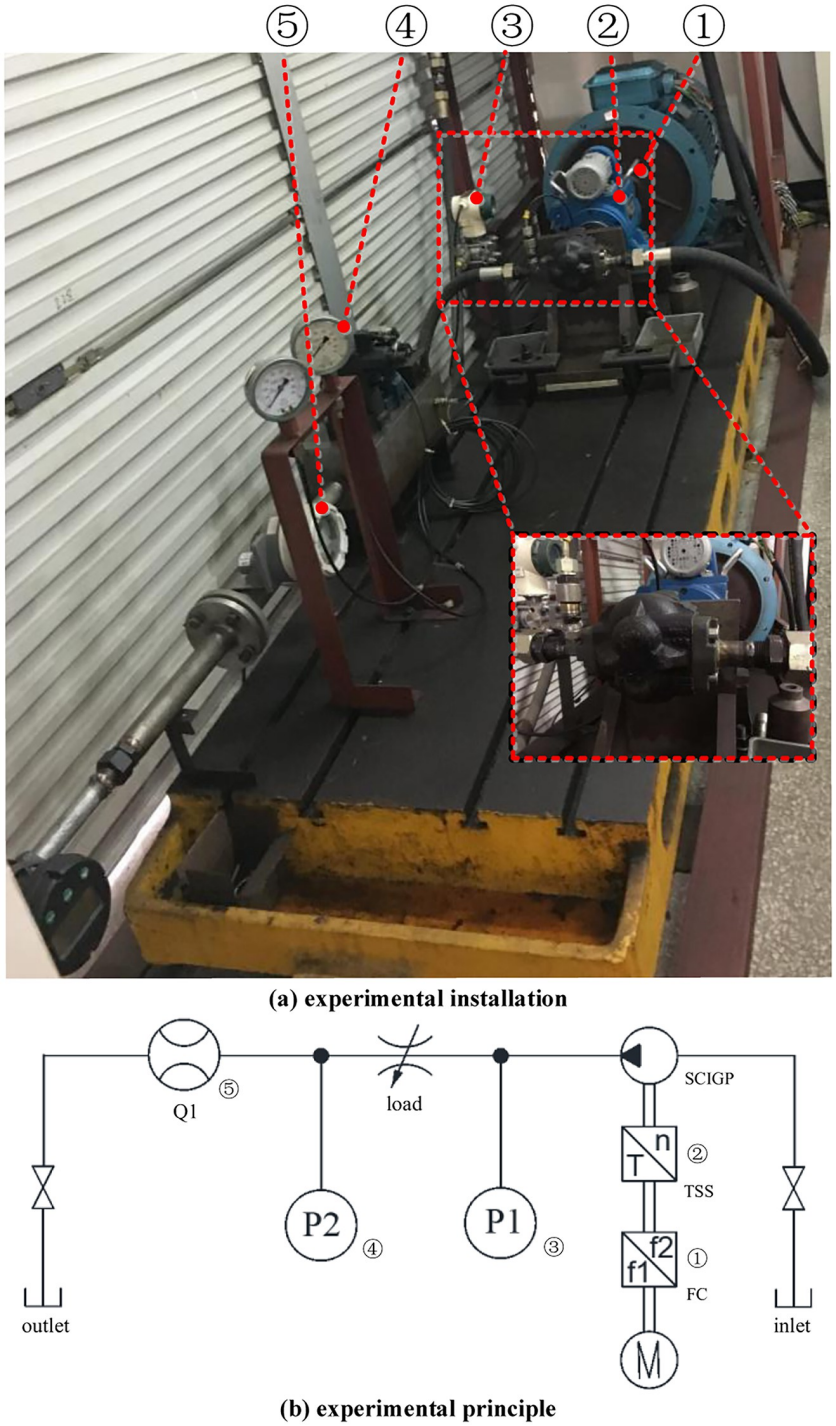
Test system. (a) experimental installation. (b) experimental principle.

The prototype of redesigned working clearances is tested on the high pressure test bench, and the corresponding test system is shown in Fig 18.

The corresponding sensor information in Fig 18 is shown in Table 14 below.

**Table 14:** Sensor information.

Name	Sensor type	Model	Main features
P1	pressure sensor	FB3351GP0S22M3	for measuring instantaneous pressure measuring range: 0–1.125KPa-41.37MPa accuracy: (including hysteresis and repeatability) ≤0.075%
P2	pressure sensor	YN-100-IV	a for detecting static pressure accuracy level: 1.5 measuring range: 0-25MP
Q1	flow sensors	FBLZJ-40-165J0	gear flow meter for detecting steady state flow measurement accuracy: liquid: class 1.5 measuring range: liquid: 0.7–10 m/s nominal pressure: 1.6–40 MPa
TSS	torque speed sensor	JC2C	real-time detection of torque and speed, for measuring input power torque measurement accuracy: 0.2 level speed measurement accuracy: ±1r/min rated torque: 2000 N.m speed range: 0–4000 r/min
FC	frequency converter	PR-F740-55K-CH	for changing pump shaft speed adapted motor power: 55 KW measurement accuracy: 0.01 Hz measuring range: 0–400 Hz

**Fig 19:**
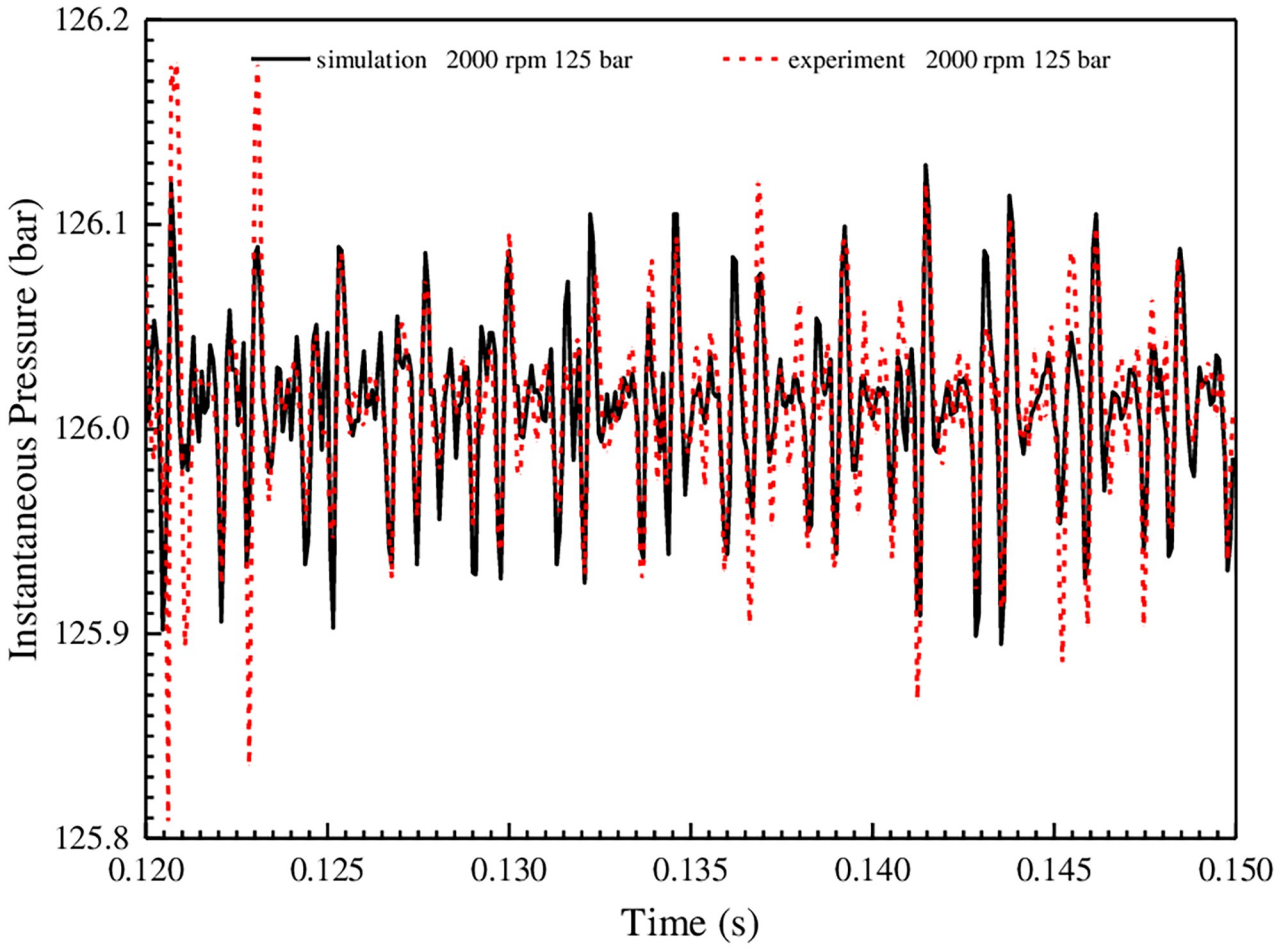
Comparison of instantaneous outlet pressure between experiment and simulation at 12.5 MPa and 2000 r/min.

In the process of the experiment, the hydraulic energy is lost by adjusting the opening of the variable throttle orifice so as to increase the oil temperature until it reaches 50°C. The instantaneous pressure at the pump outlet is measured at this temperature and compared with the simulation value, and the results are shown in Fig 19.

Fig 19 shows that when the external gear speed is 2000 r/min and the outlet pressure is 12.5 MPa, the measured value of the instantaneous outlet pressure is very close to the simulation value, and the maximum difference less than 0.01 MPa. The consistency of the experimental and simulation results verifies the applicability of the simulation model and the correctness of the simulation method.

**Fig 20:**
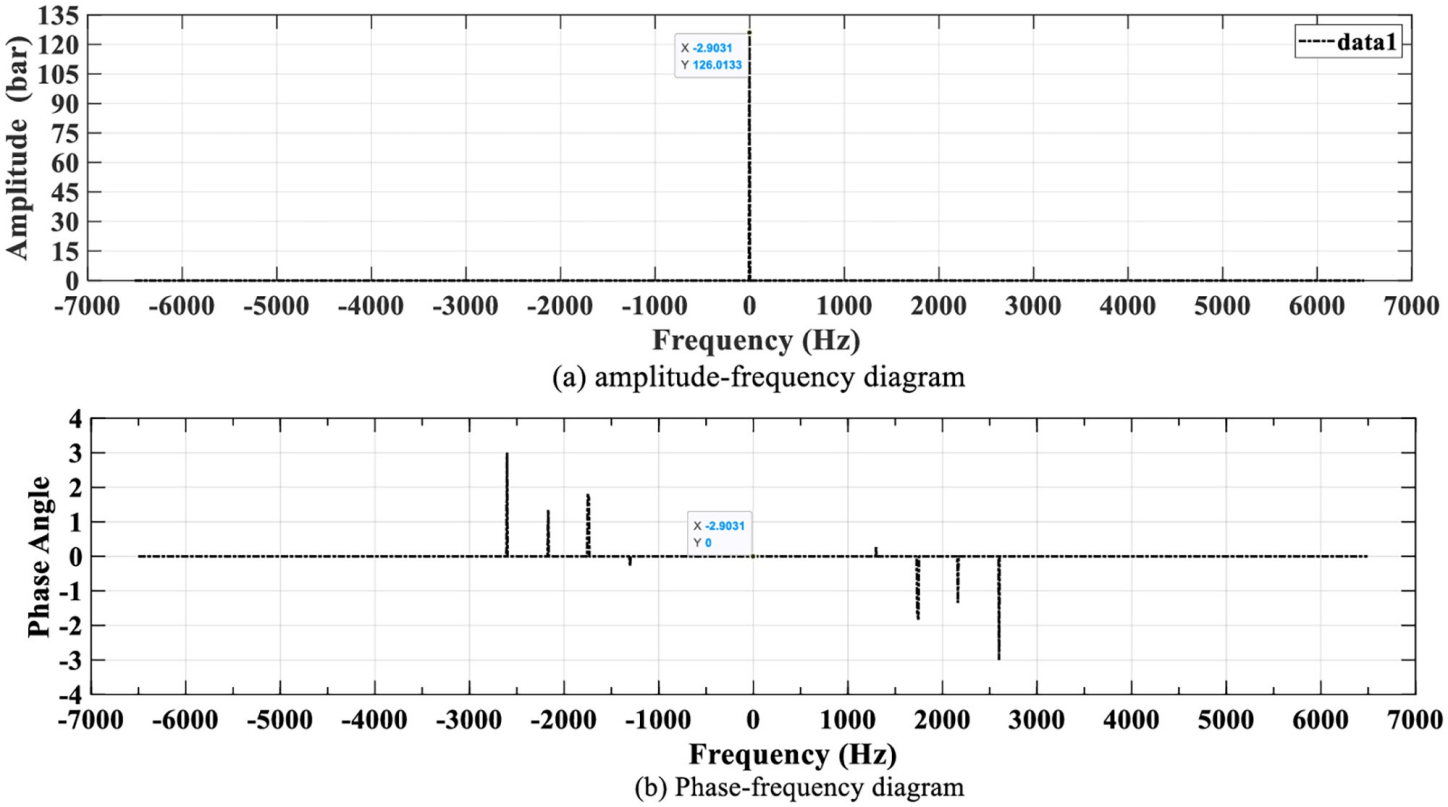
Spectrogram of simulation instantaneous pressure. (a) amplitude-frequency diagram and (b) Phase-frequency diagram.

In order to further verify the periodicity of the instantaneous pressure, the FFT full spectrum of the frequency domain signal is established, as shown in Fig 20. In addition, since the spectrums of the experiment and simulation instantaneous pressures are the same, the simulation pressure is taken as an example.

Fig 20 shows that there is only one peak point in the amplitude-frequency diagram (a), and the corresponding amplitudes of the other frequency points are all 0. The frequency of the peak point is -2.9031 Hz and the amplitude is 126.0133 L/min.

The phase frequency diagram (b) contains 8 peak points, and the phase angle at a frequency of -2.9031 Hz is 0. According to the principle of FFT, the time domain signal corresponding to the instantaneous pressure can be procured.

$$x\left(t\right)=126.0133\;\text{cos}\;2\pi f_{1}t$$ (26)

Where *f*_1_ = −2.9031.

Therefore, it can be procured that the instantaneous pressure of simulation (or experiment) is a periodic real number sequence, and the change frequency is 2.9031 Hz.

**Fig 21:**
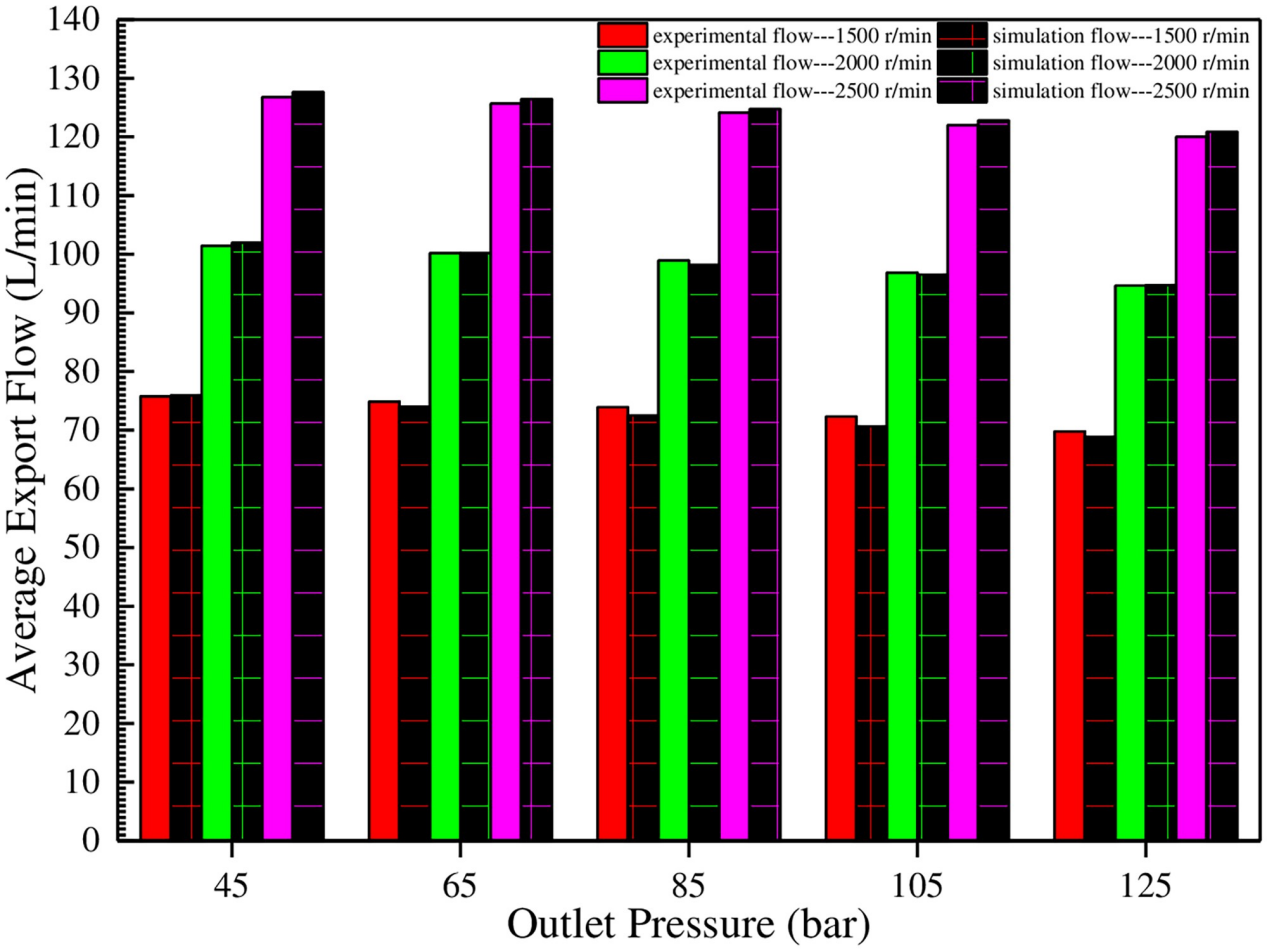
Comparison of experimental and simulated average export flows under variable working conditions.

**Fig 22:**
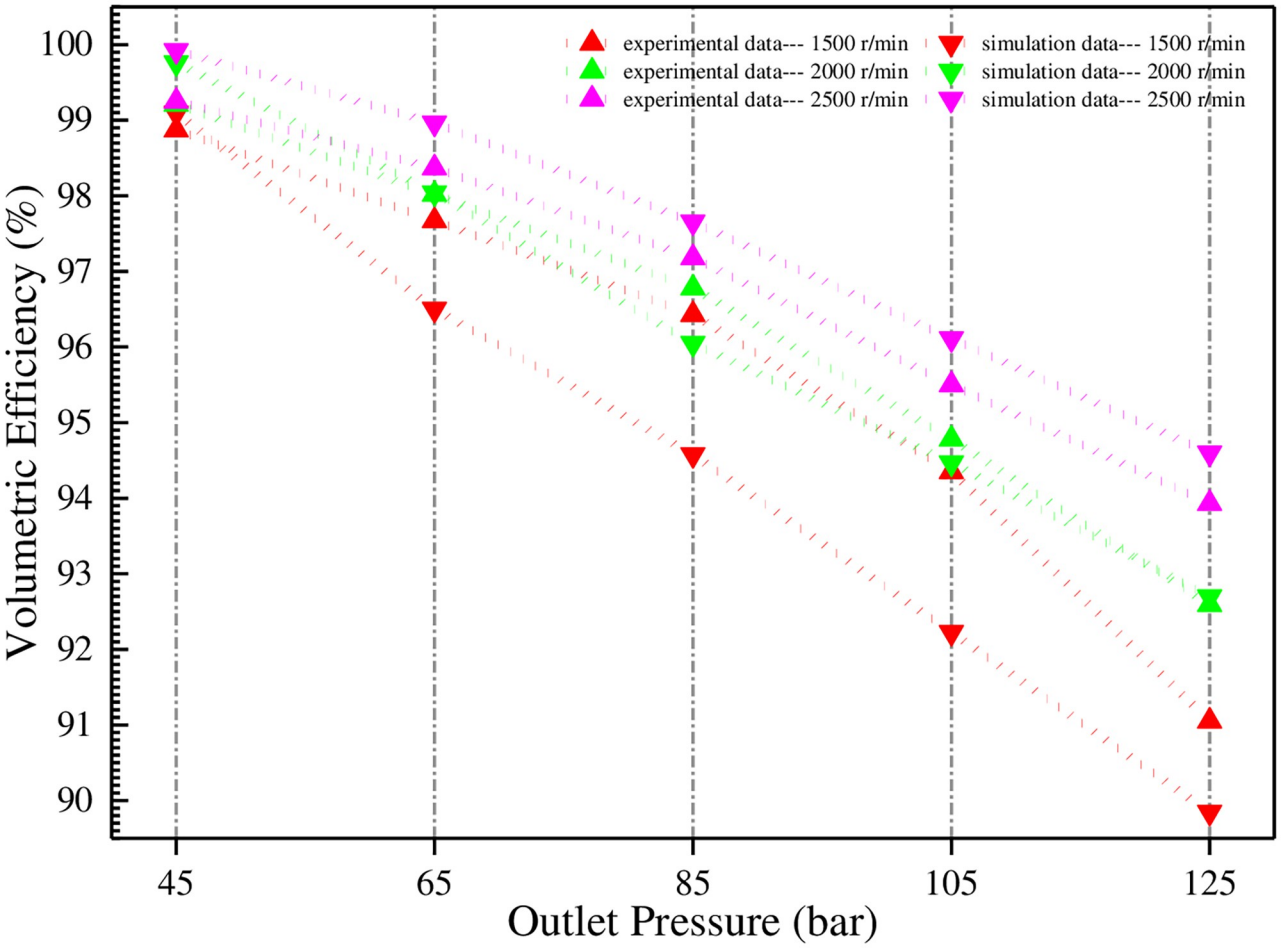
Comparison of experimental and simulated volumetric efficiencies under variable working conditions.

Next, the output flows under these conditions are measured (the speed is 1500 r/min, and the outlet pressures are 4.5 MPa, 6.5 MPa, 8.5 MPa, 10.5 MPa, and 12.5 MPa respectively; the speed is 2000 r/min, and the outlet pressures are 4.5 MPa, 6.5 MPa, 8.5 MPa, 10.5 MPa and 12.5 MPa respectively; the speed is 2500 r/min, and the outlet pressures are 4.5 MPa, 6.5 MPa, 8.5 MPa, 10.5 MPa and 12.5 MPa respectively.) respectively, and the corresponding volumetric efficiencies are calculated. The results are shown in Figs 21 and 22, respectively.

The above two figures show that when the speed is 1500 r/min, with the increase of outlet pressure, the experimental volumetric efficiency is significantly higher than the simulation, and the maximum difference between the two is about 2%. When the speed is 2000 r/min, the two are very close. When the speed is 2500 r/min, the simulation volumetric efficiency is slightly higher than the experiment. There are many reasons for this. Firstly, the gas content of the oil cannot be accurately obtained in the experiment. Secondly, the oil temperature in the system is not evenly distributed, and the local temperature may be much lower than 50°C. Thirdly, the center distance of the gear pair undergoes a slight movement after pressure-bearing, which will change the originally small working clearances. Finally, there are some unknown reasons, such as processing and assembly. Fig 22 also shows that under different speeds, with the increase of outlet pressure, the volumetric efficiency decreases parabolically. Under the same pressure, the volumetric efficiency increases with the increase of speed. This result is completely consistent with the theoretical part.

Therefore, in order to improve volumetric efficiency, the higher engine speed should be adopted as far as possible, and the pump outlet pressure should be reduced.

**Fig 23:**
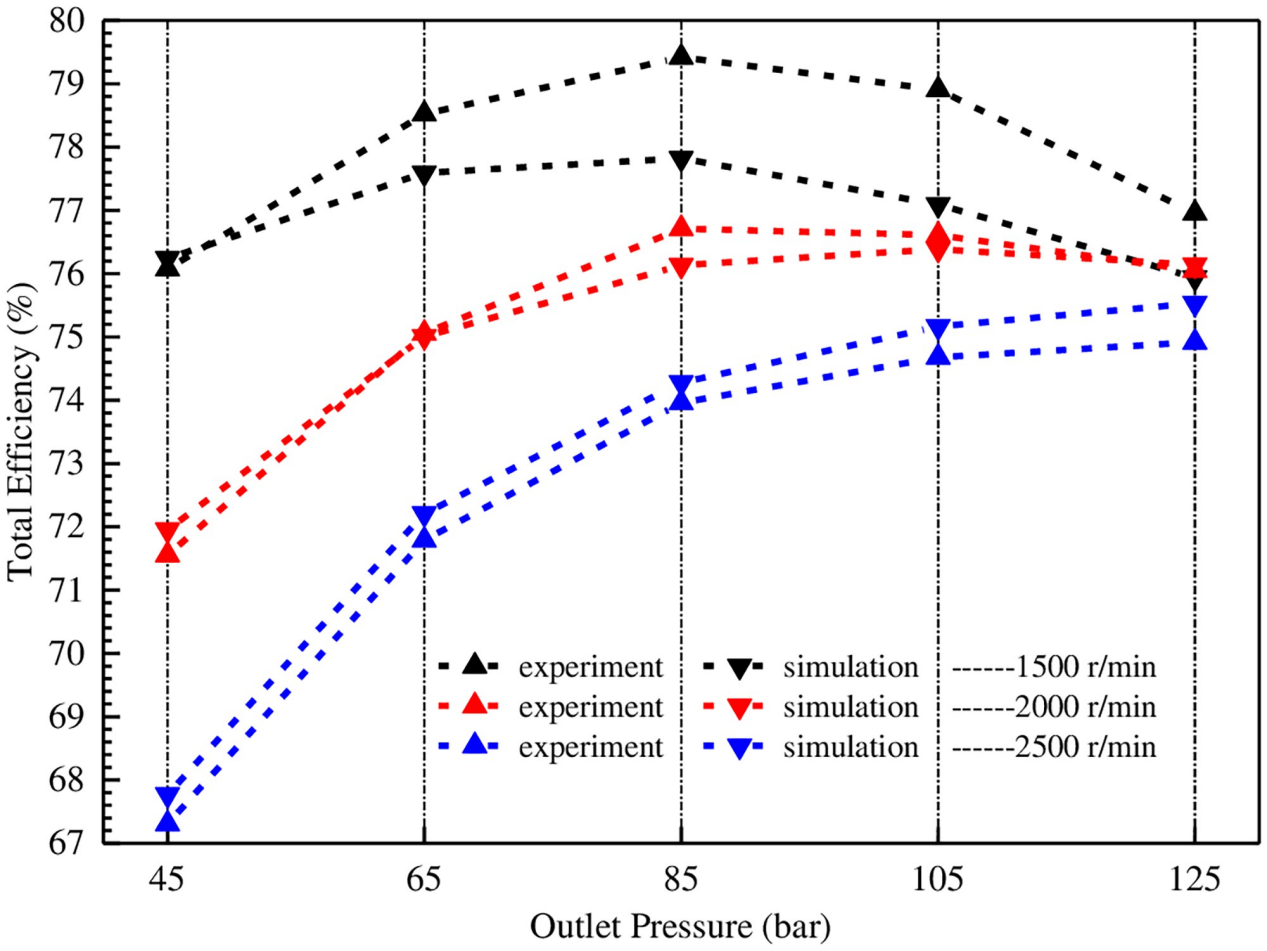
Comparison of total efficiency between experiment and simulation under variable working conditions.

Finally, the correlative input powers are measured respectively, and the homologous total efficiencies are calculated. The results are shown in Fig 23.

Fig 23 shows that when the speeds are 2000 r/min and 2500 r/min respectively, the experimental and simulated values are very close. When the speed is 1500 r/min, the experimental value is slightly larger than the simulation value, which is mainly caused by the volumetric efficiency. When the speeds are 1500 r/min and 2000 r/min respectively, the total efficiency increases first and then decreases with the increase of pressure. When the speed is 2500 r/min, the total efficiency increases with the increase of pressure. Under the same outlet pressure, the higher the speed, the lower the total efficiency. The reason is that the added value of viscous friction loss is much larger than the reduced value of leakage flow. The lower the pressure, the more obvious this degree (e.g., when the outlet pressure is 4.5 MPa, the total efficiencies corresponding to different speeds vary greatly; With the increase of pressure, this difference becomes smaller and smaller. When it reaches 12.5 MPa, the total efficiency is very close.).

Therefore, in order to improve the total efficiency, the lower the engine speeds (preferably around 1500 r/min), the better, and the pump outlet pressure is infinitely close to 8.5 MPa.

## 5. Conclusion

In this article, an orthogonal test scheme is designed to determine the optimum working clearances of the SCIGP. This paper first reviews the structural characteristics of the SCIGP. There is no automatic compensation structure for axial and radial clearances inside the pump. The friction pairs depend on the fixed small clearances to achieve sealing, lubrication and transmission of force.

The paper then describes the geometric modeling process of the SCIGP. The characteristic equation of hydrostatic support shows that when the oil film thickness is between 0.01 mm and 0.04 mm, the new oil film with different structural parameters is very conducive to adapting to the change of external load. By analyzing the mathematical models of internal leakage and viscous friction loss, it can be known that the greater the pressure difference, the smaller the viscosity, and the higher the leakage flow. The higher the speed, the lower the leakage flow is. The higher the speed, the greater the viscosity, the greater the pressure difference, the greater the viscous wear.

After that, the paper carried out numerical simulation based on the orthogonal experiment scheme. The results show that the trend of the pulsating pressure and flow curves is completely consistent, and the pressure pulsation is positively correlated with the flow pulsation. The influence of the working clearances on the pressure pulsation and the trapped oil pressure can be ignored. The three main factors affecting the flow pulsation rate, the volumetric efficiency and the total efficiency are the oil film thickness on the external gear addendum, the oil film thickness on the end face and the oil film thickness on the internal gear ring addendum, respectively. The optimal clearances can be procured by integrating various factors, that is, the oil film thickness on the end face is 0.01 mm, the oil film thicknesses on the external gear addendum and on the outer wall of internal gear ring are both 0.02 mm, the oil film thickness on the internal gear ring addendum is 0.04 mm, and the oil film thickness on the inner wall of external gear is 0.03 mm. The flow pulsation rate corresponding to the optimal clearances is 27.85%, the volumetric efficiency is 95.97%, and the total efficiency is 76.87%.

Finally, the complete prototype of the redesigned external gear and internal gear ring was tested and compared with the simulation results. The results show that the experimental pressure fluctuation curve is highly consistent with the simulation, which verifies the applicability of the simulation model and the correctness of the simulation method. Under the same pressure, the higher the speed, the greater the volumetric efficiency is, and the lower the total efficiency. In order to make the SCIGP work at the highest total efficiency, the lower engine speeds should be used (preferably around 1500 r/min), and the pump outlet pressure is infinitely close to 8.5 MPa.

In future research, the micro-motion analysis is carried out according to the force characteristics of gear pairs, and In the case of eccentricity after pressure-bearing, the value range of the working clearance of the gear pairs will be re-determined. In addition, the clearance compensation mechanism under high pressure conditions will be studied.

## Abbreviations

*A*_1_: Friction area of external gear addendum (mm^2^)
*B*: Tooth width (mm)
*d*: Inner diameter of external gear (mm)
*dA*′: Micro-ring area with the width *dr*′ (mm^2^)
*dr*′: Micro ring width (mm)
*e*: Bearing hole depth (mm)
*F_1_*: External load force (N)
*h*: Oil film thickness (mm)
*h*_1_: Oil film thickness on the end face (mm)
*h*_2_: Oil film thickness on the external gear addendum (mm)
*h*_3_: Oil film thickness on the internal gear ring addendum (mm)
*h*_4_: Oil film thickness on the inner wall of external gear (mm)
*h*_5_: Oil film thickness on the outer wall of internal gear ring (mm)
*K*: Structural parameter of hydrostatic support. For a given hydrostatic support, *K* is a constant
*k_q_*: Clearance damping leakage coefficient
*k*_*q*1_: Clearance damping flow coefficient
*n*_1_: Rotation speed of external gear (r/min)
*n*_2_: Rotation speed of internal gear ring (r/min)
*p*: Inlet pressure of damper (MPa)
*p_s_*: Pressure in damper cavity (MPa)
*Q*: Flow rate (L/min)
*r*: Inner radius of external gear (mm)
*r*_1_: Radius of external gear index circle (mm)
*r*_2_: Radius of internal gear ring index circle (mm)
*r*_a1_: Radius of external gear addendum circle (mm)
*r*_a2_: Radius of internal gear ring addendum circle (mm)
*r*_f1_: Radius of external gear dedendum circle (mm)
*r*_f2_: Radius of internal gear ring dedendum circle (mm)
$r_{1}^{\prime }$: Any radius between the inner wall of external gear and dedendum circle (mm)
*R*: Outer radius of internal gear ring (mm)
*R*_f_: Hydraulic resistance of various fixed dampers
SCIGP: Straight conjugate internal gear pumps
*s*_*e*1_: Thickness of external gear addendum (mm)
*s*_*e*2_: Thickness of internal gear ring addendum (mm)
*u*_1_: Total leakage velocity between external gear addendum and crescent diaphragm (m/s)
*v*_1_: Linear velocity of oil at the radius $r_{1}^{\prime }$(m/s)
*v*_a1_: Linear velocity of external gear addendum (m/s)
*y*: Any thickness between the external gear addendum and the crescent plate (mm)
$z_{1}^{\prime }$: Number of external gear teeth involved in the top seal
$z_{2}^{\prime }$: Number of internal gear ring teeth involved in the top seal
*α*: Dimensionless pressure ratio
*β*_1*h*_: Wrap angle of high pressure cavity corresponding to external gear (°)
*β*_2*h*_: Wrap angle of high pressure cavity corresponding to internal gear ring (°)
2*β*_1*t*_: Wrap angle of transition cavity corresponding to external gear (°)
2*β*_2*t*_: Wrap angle of transition cavity corresponding to internal gear ring (°)
Δ*N*: Total viscous friction loss inside gear pump (KW)
Δ*N*_1_: Axial clearance power loss (KW)
Δ*N*_2_: Radial clearance power loss (KW)
Δ*N*_f1_: Viscous friction loss in the end face clearance of external gear (KW)
Δ*N*_f2_: Viscous friction loss in the end face clearance of internal ring gear (KW)
$\Delta N_{\text{f1}}^{\prime }$: Viscous friction loss on the unilateral end face from gear shaft to dedendum circle (KW)
$\Delta N_{\text{f2}}^{\prime \prime }$: Viscous friction loss on the unilateral end face from dedendum circle to addendum circle (KW)
Δ*N*_*h*2_: Oil viscous friction loss on the external gear addendum surface (KW)
Δ*N*_*h*3_: Oil viscous friction loss on the internal gear ring addendum surface (KW)
Δ*N*_*h*5_: Viscous friction loss on the outer wall of internal gear ring (KW)
Δ*p*: Pressure difference between oil discharge and oil suction cavities (MPa)
Δ*p*′: Pressure difference between inlet and outlet of fixed damping (MPa)
Δ*p*″: Pressure difference between inlet and outlet of variable damping (MPa)
Δ*q*: Total leakage flow inside gear pump (L/min)
Δ*q*_1_: Total leakage in the gear end face clearance (L/min)
Δ*q*_2_: Radial clearance leakage flow (L/min)
Δ*q*_*h*2_: Leakage flow between external gear addendum and crescent diaphragm (L/min)
Δ*q*_*h*3_: Leakage flow between internal gear ring addendum and crescent diaphragm (L/min)
Δ*q*_*h*4_: Leakage flow between the inner wall of external gear and the bearing (L/min)
Δ*q*_*h*5_: Leakage flow between the outer wall of internal gear ring and the inner wall of the shell (L/min)
*ε*: Eccentricity
*μ*: Dynamic viscosity (Pa.s)
*τ*_*h*1_: Friction shear stress of oil on the end face of external gear (Pa)
*τ*_*h*2_: Friction shear stress on the external gear addendum surface (Pa)
*ω*: Angular speed of external gear (rad/s)
